# Immunomodulators in Graves’ ophthalmopathy: a systematic review

**DOI:** 10.3389/fendo.2026.1686786

**Published:** 2026-02-10

**Authors:** Antoinette Yeung, Mohammed Saqlain Siddiqui, Neginsadat Mirtorabi, Umnia Nasir Ahmed, Emma Watts, Neil Sharma, Reena Kumari, Kristien Boelaert, Jameel Muzaffar, Hannah Nieto

**Affiliations:** 1 Ear, Nose and Throat Surgery, University Hospitals Birmingham National Health Service (NHS) Foundation Trust, Birmingham, United Kingdom; 2 National Health Service, University of Birmingham Medical School, Birmingham, United Kingdom; 3 Ophthalmology, University Hospitals Birmingham National Health Service (NHS) Foundation Trust, Birmingham, United Kingdom; 4 Department of Applied Health Sciences, University of Birmingham, Birmingham, United Kingdom; 5 Department of Metabolism and Systems Science, University of Birmingham, Birmingham, United Kingdom

**Keywords:** Graves, ophthalmopathy, immunomodulators, clinical activity score, proptosis, orbitopathy

## Abstract

**Background:**

Graves’ Ophthalmopathy (GO), or thyroid eye disease, is an extrathyroidal complication of Graves’ disease, causing a significant impact on patients’ quality of life. There are consensus statements from both the American Thyroid Association (ATA) and the European Group on Graves’ Orbitopathy (EUGOGO) which provide guidance in management. However, there remain areas of ongoing debate and evolution in treatment approaches. This systematic review aims to evaluate immunomodulatory drugs in the treatment of GO.

**Methods:**

The following databases were searched: Cochrane (CENTRAL), Embase, MEDLINE, ProQuest, PubMed, SCOPUS, and Web of Science. The last date of searching for each database was 10th October 2025. All primary studies on human patients with GO, treated with newer, targeted immunomodulatory therapies, in particular biologics and/or disease-modifying antirheumatic drugs, were included. Studies in languages other than English were excluded. The primary outcome of interest was Clinical Activity Score (CAS). A narrative synthesis was performed, including CAS and four other secondary outcomes. Cochrane risk of bias tools were used to assess quality of evidence. The study is registered on PROSPERO (CRD42023400285).

**Results:**

Of 4839 records identified, 41 were eligible for data extraction. Teprotumumab and tocilizumab showed benefit in reducing CAS and proptosis, whilst rituximab was inconclusive. Methotrexate and reduced-dose steroids were beneficial in reducing CAS, and both methotrexate and cyclosporine with steroids were good for reducing proptosis. However, effects of immunomodulation on other secondary outcomes were unclear. Adverse events were higher in the steroid treated patients compared to all other drugs.

**Conclusion:**

Biologics and steroid-sparing agents may be more effective than steroids at reducing CAS, but this is limited by the lack of head-to-head comparisons between drugs and significant heterogeneity amongst the included studies.

**Systematic Review Registration:**

https://www.crd.york.ac.uk/prospero/, identifier CRD42023400285.

## Introduction

Graves’ ophthalmopathy (GO) is a potentially sight-threatening autoimmune disease, characterized by inflammation of the orbit and its surrounding tissues. It forms the most frequent extrathyroidal manifestation of Graves’ disease (1). At least 50% of patients with Graves’ disease develop clinically significant GO (2), leading to an estimated prevalence in Europe of 90–155 per 100,000 people (3). The most recent guidelines from the European Group on Graves’ Orbitopathy (EUGOGO) subcategorizes presentation as mild, moderate-to-severe, or sight-threatening disease (4). GO can be subclassified further into inactive and active phases of GO.

Typically, diagnosis relies on the presence of key clinical features on a background of confirmed Graves’ disease (5). However, thyroid stimulating hormone receptor antibodies (TRAbs) have demonstrated independent association with GO and the potential to predict disease severity and clinical outcomes (6). Currently, TRAbs are the only biomarker specific to both Graves’ disease and GO (7).

Distinguishing active and inactive GO is currently measured using the validated Clinical Activity Score (CAS), which helps direct treatment strategies (**Table 1**) (4, 9).

**Table 1:** - CAS score.

For initial CAS score items 1-7
1) Spontaneous orbital pain
2) Gaze evoked orbital pain
3) Eyelid swelling that is considered to be due to active GO
4) Eyelid erythema
5) Conjunctival redness considered due to active GO
6) Chemosis
7) Inflammation of caruncle or plica

**Table 1**. Clinical Activity Score (CAS) (8). CAS is a scoring system that is used to indicate the level of activity of Graves’ Ophthalmopathy. One point is given to each item; a score of ≥3/7 is indicative of active disease (9). Table taken from: https://doi.org/10.1136/bjo.73.8.639 (10). GO: Graves’ Ophthalmopathy.

Historically, corticosteroid therapy was the mainstay of active, moderate-to-severe GO (11) and remains popular for alleviating orbital inflammation (8, 12). Other interventions include orbital radiotherapy and corticosteroid-sparing agents such as methotrexate and cyclosporine (13, 14). These are all associated with significant side effects and can be quite co-morbid, so alternative treatments are welcome.

Recent developments in our understanding of the immunological basis of GO have placed increasing focus on newer, more targeted immunomodulatory therapies such as monoclonal antibodies. In 2020, the monoclonal antibody teprotumumab became the first FDA approved drug for the treatment of GO in adult patients (15).

The EUGOGO 2021 guidelines outline clear treatment recommendations for mild disease and first-line strategies for moderate-to-severe disease (4). The evidence basis for second-line treatments in moderate-to-severe disease is less prescriptive due to a lack of sufficient evidence. As newer treatment strategies emerge and our understanding of existing medications expands, uncertainty grows regarding the optimal management of complex GO.

This systematic review aims to analyze the current evidence regarding immunomodulation in GO to determine the most effective treatment strategies.

## Methods

The review was conducted in accordance with PRISMA guidelines (Appendix A) (16). The study protocol was prospectively registered on the PROSPERO database of systematic reviews (CRD42023400285).

A search strategy was developed in collaboration with information specialist librarians [JD, EJ] (Appendix B). Published records were collected by searching systematically on Cochrane (CENTRAL), Embase, MEDLINE, ProQuest, PubMed, SCOPUS, and Web of Science databases with no limits placed on date. Reference lists from the selected studies were assessed against the eligibility criteria (**Table 1**). Case reports, literature reviews and non-English language papers were excluded. Titles and abstracts were independently screened by two blinded reviewers [SY, SS]. All discrepancies were resolved through discussion with an independent reviewer [JM]. The PICO framework is documented in (**Table 2**).

**Table 2:** – PICO framework.

Population	Adults (≥18 years) with GO
Interventions	Targeted immunomodulatory therapies (especially biologics ± DMARDS)
Comparator	Current treatments for GO
Outcomes	Primary Outcome: Clinical Activity Score Secondary Outcome: Proptosis, TRAb reduction, B Cell Response, QOL, Adverse Events

Data extraction was performed by two reviewers [AY, SS]. Data were collected and entered into a standardized spreadsheet for analysis. Patient characteristics were reviewed to ensure comparability between studies. Our primary data outcome was CAS. However, where data was not available, alternative scoring methods were extracted, including assessment of TRAb reduction and B cell depletion. Specifically generated composite indices were also included, provided they were supported by an appropriately cited evidence base.

The Cochrane RoB (Risk of Bias) 2 tool (17) was used for randomized trials, whilst non-randomised studies were assessed with the Cochrane ROBINS-I (Risk of Bias in Non-randomized Studies – of Interventions) tool (18). Data was presented using the Robvis tool (19). The risk of bias assessments were conducted by two reviewers [AY, SS] and the same risk of bias tools were used to assess bias in missing data.

## Results

**Figure 1:**
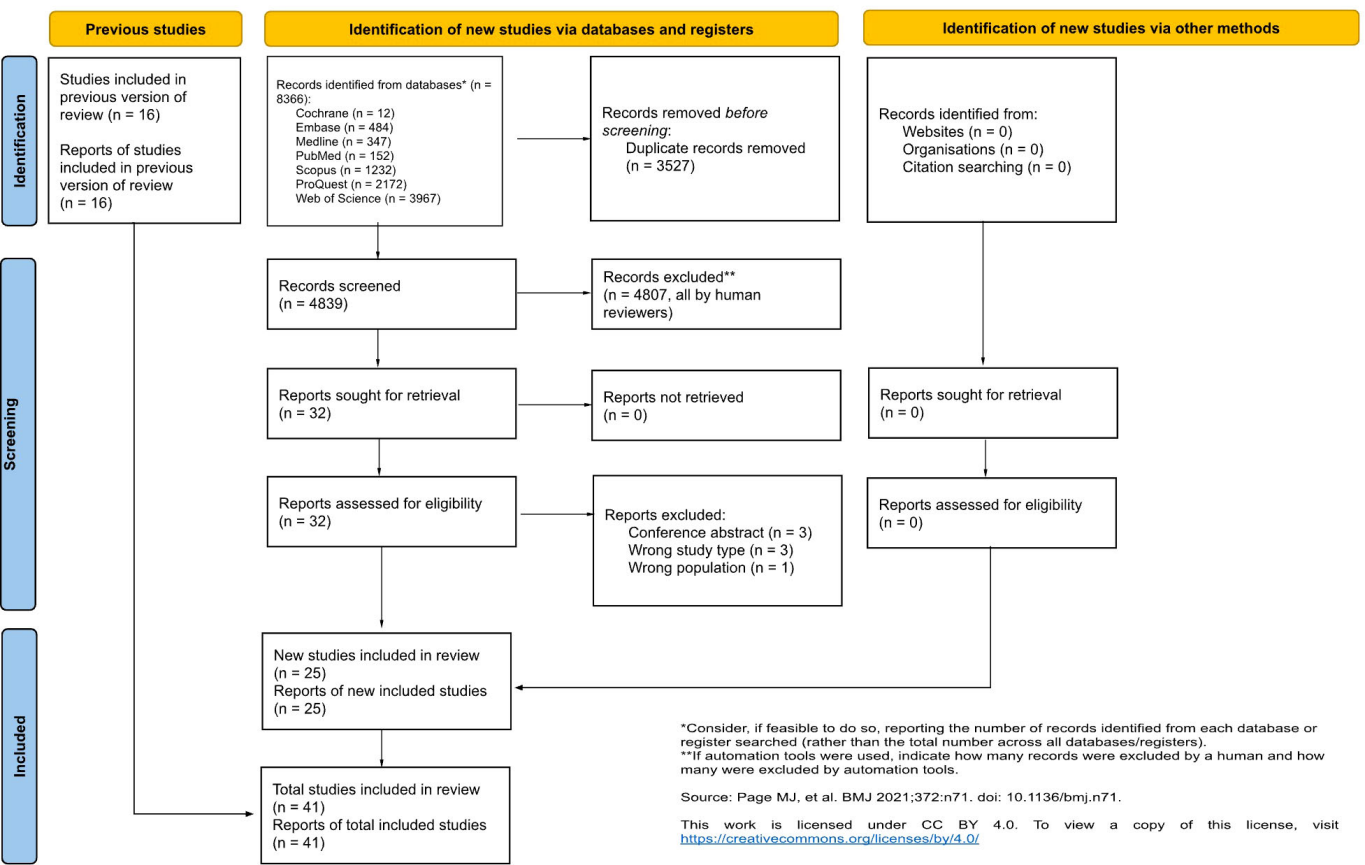
PRISMA Flowchart.

Our search strategy identified 4839 records across all seven databases after removal of duplicates. After screening, 41 studies met criteria for inclusion; fourteen randomized controlled trials, four prospective cohort studies, eleven retrospective cohort studies and twelve case series. Reasons for report exclusion are documented in (**Figure 1**).

(**Table 3**) presents the characteristics of all 41 studies included in this systematic review. These studies comprise 1546 participants in total. Heterogeneity in outcome measurement, type of drug treatment, drug dosing and duration of treatment is present across all studies. Previous thyroid treatment also varied greatly across all studies. Patient characteristics are presented in (**Supplementary Table 5**).

**Table 3:** - Study characteristics.

Study reference	Type of Study	No. of patients	Intervention	Dosage of drug(s)	Dosage time frame	Main outcome	Secondary outcome	Mean follow-up (months)
Antonelli et al., 1992 (20)	RCT	26	Group 1: IVIG + OR	IVIG: 400mg/kg	5 days repeated 3x every 3 weeks	Ophthalmopathy index Proptosis	–	6
			Group 2: IVIG	MP: 80mg	2 weeks			
			Group 3: MP + OR	OR: 200cGy	2 weeks			
Utech et al., 1995 (21)	Prospective case series	39	Cyclosporine	2.5mg/kg	Mean 27 weeks	MRI T2 relaxation time	Muscle thickness IOP	12, 36, 72
			Prednisolone	60mg	3 months			
Kahaly et al., 1996 (22)	RCT	40	IVIG	1g/kg	2 days repeated 6x every 3 weeks	Proptosis	–	4.6
			Prednisolone	100mg/day				
Ozata et al., 1996 (23)	Prospective case series	10	Octreotide	100mcg	3 months	sICAM-1	Proptosis Ophthalmopathy index	3
Dickinson et al., 2004 (24)	RCT	50	Octreotide Long Acting Repeatable (LAR-LAR)	30mg every 4 weeks	32 weeks	Ophthalmopathy Index (OI) score	CAS Proptosis IOP GO-QOL Orbital muscle and fat volumes by MRI Palpable aperture Diplopia	13
			Placebo-LAR	0.9% sodium chloride then 30mg every 4 weeks	16 weeks then 16 weeks			
Meyer, 2006 (25)	Retrospective case series	14	IVMP	10mg/kg	3x every 2 days	Visual acuity Visual fields Ocular motility	AEs	36
			Prednisolone	30mg	2 years			
			Cyclosporine	2mg/kg	2x for 2 days			
Stan et al., 2006 (26)	RCT	25	Octreotide Long Acting Repeatable (LAR)	20mg every 4 weeks	4 months	CAS	Retrobulbar tissue volume Lid fissure width Range of motion Diplopia	4
			Placebo	0.9% saline	4 months			
Salvi et al., 2008 (27)	Prospective cohort study	29	IV RTX	2x 1g	2 weeks apart	CAS NOSPECS	Thyroid function B cell count	12
			IVGC	500mg	16 weeks			
Khanna et al., 2010 (28)	Retrospective case series	6	RTX	2x 1g	2 weeks apart	CAS Proptosis Strabismus AEs T_reg_ cells	–	6.2
Silkiss et al., 2010 (29)	Prospective case series	12	RTX	2x 1g	2 weeks apart	CAS TAOS	B cell count, TSI, TSH	12
Tambe et al., 2010 (30)	Retrospective case series	36	IVMP	1g	3 days	Efficacy of IVMP	Proptosis Optic nerve function Extraocular muscle movement	0.92
Mitchell et al., 2013 (31)	Retrospective case series	9	RTX	3 pts - 2x 2g	2 weeks apart	CAS B cell count TFTs TRAb NOSPECS	–	12
				6 pts - 2x 1g	2 weeks apart			
Perez-Moreiras et al., 2014 (32)	Prospective interventional non- randomised study	18	Tocilizumab	8mg/kg once a month	Until CAS <1 or TSI negative	CAS TSI levels	Ocular motility Diplopia Proptosis	9
Stan et al., 2015 (33)	RCT	21	RTX	2x 1g	2 weeks apart	CAS	Proptosis QOL Lid fissure width Diplopia Lagophthalmos Disease severity	12
			Placebo	0.9% sodium chloride				
Smith et al., 2017 (34)	RCT	88	Teprotumumab	8 infusions: 10mg/kg then 20mg/kg	24 weeks	CAS Proptosis	QOL	11
			Placebo	0.9% sodium chloride				
Perez-Moreiras et al., 2018 (35)	RCT	32	Tocilizumab	8mg/kg once a month	4 months	CAS	QOL Anti-TPO TSH TSI Disease severity	10
			Placebo	0.9% sodium chloride				
Deltour et al., 2018 (36)	Retrospective cohort study	40	RTX	2x 1g	2 weeks apart	CAS Oculomotor dysfunction Visual dysfunction	Proptosis Visual acuity Diplopia	6
Insull et al., 2019 (37)	Retrospective case series	12	RTX	100mg	1 hour	CAS	VISA Ox-TED QoL TRAb B cell count AEs	6.3
Douglas et al., 2020 (38)	RCT	83	Teprotumumab	8 infusions: 10mg/kg then 20mg/kg	24 weeks	Proptosis	Overall response (CAS + proptosis) Diplopia response GO-QOL	6
			Placebo	–				
Ceballos-Macias et al., 2020 (39)	Retrospective case series	8	Tocilizumab	8mg/kg once a month	6 months	CAS Proptosis TRAb levels	–	6
Perez-Moreiras et al., 2021 (40)	Retrospective Longitudinal Study	54	Tocilizumab	8mg/kg once a month	4 months	CAS TRAb levels Proptosis Diplopia		22
Smith et al., 2021 (41)	Retrospective case series	9	Tocilizumab	8mg/kg once a month	4 months	CAS TAOS TSI	TAOS TSI	24
Moi et al., 2021 (42)	Retrospective cohort study	10	Tocilizumab	6 months: 8mg/kg once a month 6 months: 162mg subcut OW 6 months: 162mg subcut fortnightly	18 months	CAS Proptosis Diplopia TRAb	–	6
Ugradar et al., 2022 (43)	Retrospective cohort study	31	Teprotumumab	8 infusions: 10mg/kg then 20mg/kg	24 weeks	Proptosis	Diplopia CAS Dysmotility Fat and muscle volume reduction	6
Ozello et al., 2022 (44)	Retrospective case series	9	Teprotumumab	8 infusions: 10mg/kg then 20mg/kg	24 weeks	Proptosis Eyelid retraction	Adverse events Surgery post treatment	17
Douglas et al., 2022 (45)	RCT	51	Teprotumumab	8 infusions: 10mg/kg then 20mg/kg	24 weeks	Proptosis AEs	CAS Diplopia QoL	11
Bennedjai et al., 2022 (46)	Comparative Retrospective Cohort Study	21	Tocilizumab	8mg/kg once a month	4 months	CAS	Proptosis Visual acuity Diplopia Chemosis Eye aperture Relapse	11
			Rituximab	2x 100mg	2 weeks apart			16
Pan et al., 2022 (47)	RCT	100	Doxycycline	50mg	12 weeks	Eyelid aperture QOL Proptosis Ocular motility	CAS Upper eyelid lag Eyelid retraction Ocular Surface Disease Index	2.8
			Placebo	Starch	12 weeks			
Shen et al., 2022 (48)	RCT	90	Group 1: MP	MP=IV 0.5g/week then 0.25g/week	6 weeks 6 weeks	CAS	Proptosis Diplopia IOP Visual acuity QoL AEs	2.8
			Group 2: Reduced MP + MTX	Reduced MP = IV, 0.25g/week	12 weeks			
			Group 3: full-dose MP	MTX = oral 10mg/week then 12.5mg/week	2 weeks 10 weeks			
Douglas et al., 2023 (49)	RCT	62	Teprotumumab	8 infusions: 10mg/kg then 20mg/kg	24 weeks	Proptosis	GO-QOL Diplopia	6
			Placebo	-				
Boutzios et al., 2023 (50)	Retrospective observational study	12	Tocilizumab	8mg/kg once a month	4 months	CAS	TSI levels Proptosis Diplopia	5.5
Wang et al., 2023 (51)	Retrospective Case Series	6	Rituximab	125mg/m^2^ body surface area	Once per week for 4 weeks	CAS	B-cell depletion TRAb IOP Visual acuity MRI parameters	56
Men et al., 2024 (52)	Retrospective cohort study	66	Teprotumumab	8 infusions: 10mg/kg then 20mg/kg	24 weeks	Proptosis CAS Diplopia	–	8
Hoang et al., 2024 (53)	Retrospective non comparative study	26	Teprotumumab	8 infusions: 10mg/kg then 20mg/kg	24 weeks	Proptosis	TFTs TRAb CAS IOP Extraocular motility Eyelid position	24
Rosenblatt et al., 2024 (54)	Retrospective cohort study	119	Teprotumumab	8 infusions: 10mg/kg then 20mg/kg	24 weeks	Proptosis	CAS	10.5
Matoc et al., 2024 (55)	RCT	82	Doxycycline	50mg OD	12 weeks	CAS Modified GO-QOL Ocular motility Diplopia Proptosis	MRD1 and 2 Eyelid aperture Levator palpebrae superioris function	12
			No treatment	–				
Habroosh et al., 2024 (56)	Prospect Longitudinal Cohort study	13	Tocilizumab	8mg/kg once a month	4 months	CAS Visual acuity Proptosis TSI	–	20.5
Lee et al., 2024 (57)	Prospective Cohort study	19	Tocilizumab	8mg/kg once a month	4 months	CAS NOSPECS Proptosis Extra ocular muscle limitation Diplopia	TRAb	22.8
Al-Sharif et al., 2024 (58)	Retrospective Cohort study	91	Teprotumumab	8 infusions: 10mg/kg then 20mg/kg	24 weeks	Reduction in eyelid retraction MRD1, MRD2 Proptosis	–	9
Farde et al., 2025 (59)	Retrospective Cohort study	23	Tocilizumab	8mg/kg once a month	4 Months	≥2 mm Hertel reduction	Improvement in CAS ≥ 2 points Reduction of diplopia by ≥1 point (Gorman score) TRAb levels	10
Hiromatsu et al., 2025 (60)	RCT	54	Teprotumumab	8 infusions: 10mg/kg then 20mg/kg	24 weeks	Proptosis CAS Diplopia GO-QOL	–	6
			Placebo	–	24 weeks			

**Table 3**. Study characteristics of included studies. Note that dosages were altered over the course of treatment in many studies, particularly for steroids, which were gradually tapered over several weeks or months. AEs: adverse events; CAS: clinical activity score; IOP: intraocular pressure; GO-QOL: Graves’ Ophthalmopathy Quality of Life score; IVGC: intravenous glucocorticoids; IVIG: intravenous immunoglobulin; MP: methylprednisolone; MTX: methotrexate; OR: Orbital Radiotherapy; OW: once weekly Pred: prednisolone; pts: patients; QoL: quality of life; RCT: randomised controlled trial; RTX: rituximab; TAOS: Thyroid Associated Ophthalmopathy Scale; TRAb: TSH-receptor antibody; T_reg_ cells: regulatory T cells; TSH: thyroid stimulating hormone; TSI: Thyroid stimulating immunoglobulins, TFTs: thyroid function tests, IOP: intraocular pressure.

NOSPECS and VISA are alternative clinical scoring systems to CAS.

**Figure 2:**
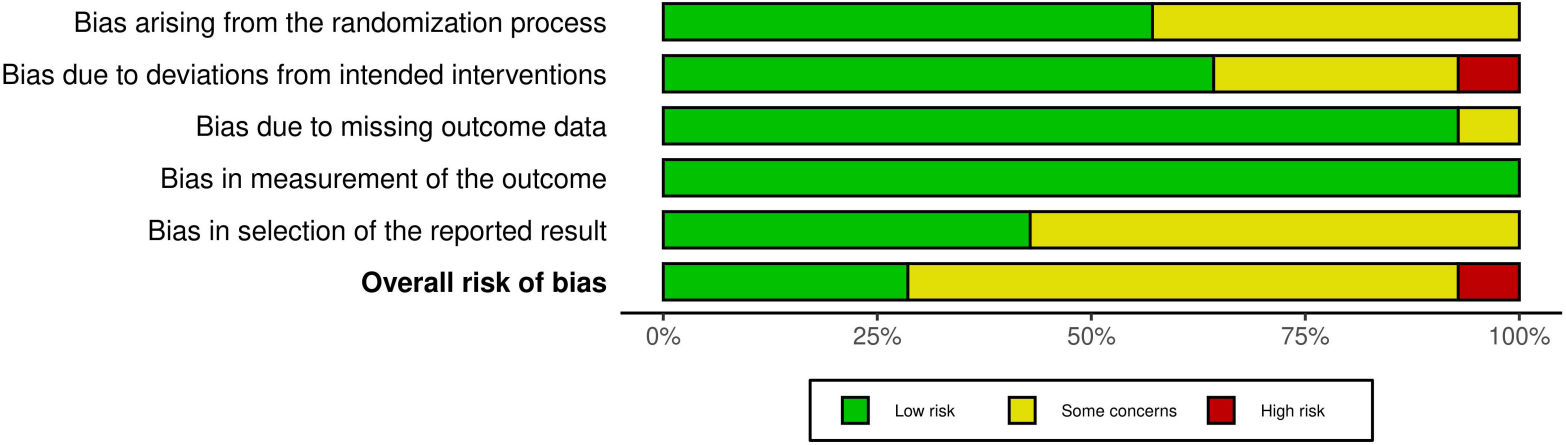
Summary plot for risk of bias for randomised trials.

**Figure 3:**
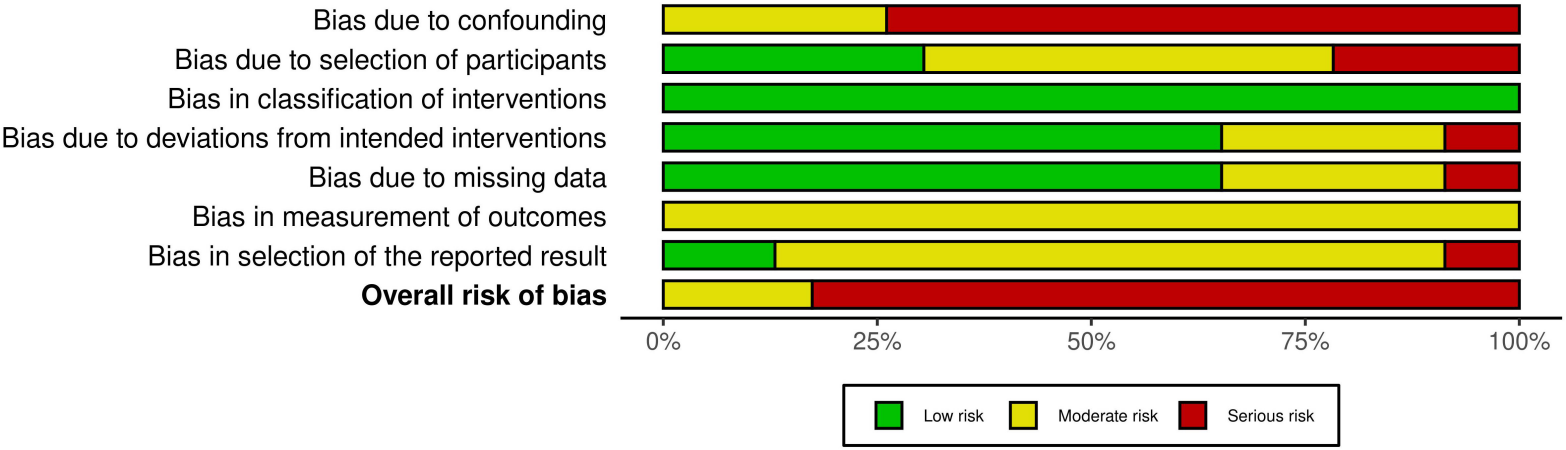
Traffic light plot of the risk of bias for randomised trials using the Robvis tool (22).

**Figure 4:**
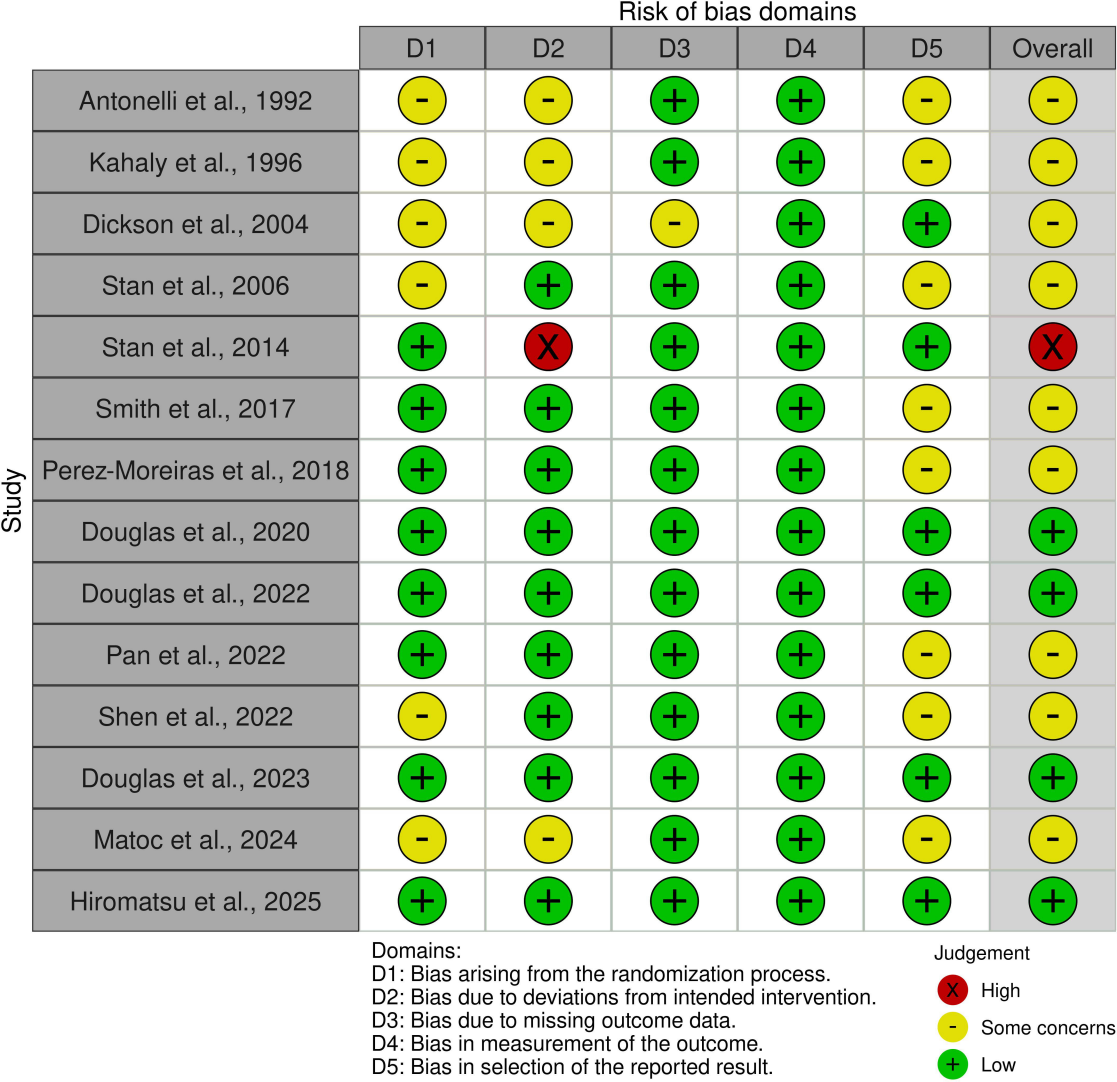
Summary plot of risk of bias for non-randomised trials.

**Figure 5:**
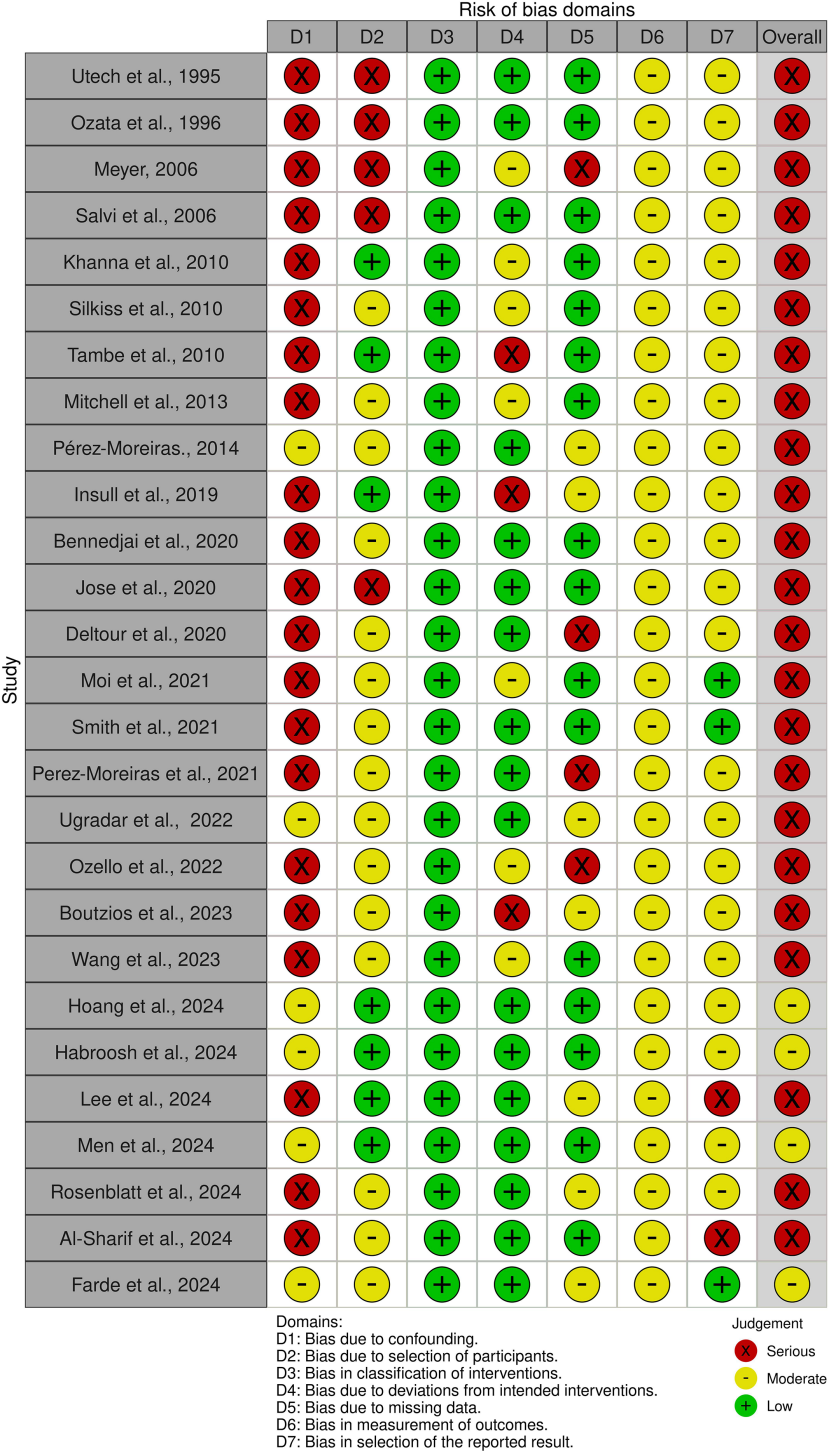
Traffic light plot of the risk of bias for non-randomised trials using the robvis tool (22).

Risk of bias was assessed for fourteen RCTs included in this review (**Figures 2**, **3****).** All were assessed as having low or some concerns for bias with the exception of Stan et al. (31) which had a serious risk of bias. Concerning non-RCT studies, most had a serious risk of bias mostly due to the lack of control variables and consideration for confounding factors (**Figures 4**, **5**).

(**Table 4**) demonstrates the CAS outcomes. 24 studies reported CAS as their main outcome and 8 studies included CAS as a secondary outcome. All studies looking at rituximab demonstrated a statistically significant decrease in mean or median CAS score after treatment apart from Stan et al. (31) which showed no significant difference compared with placebo. Similarly, teprotumumab was shown to significantly improve CAS score but did not show any significant change in Douglas et al. (41). Doxycycline demonstrated a reduction in the number of patients with a CAS score of 3 and above in Matoc et al. (49) but did not show any statistically significant changes in Pan et al. (43). Methotrexate with either reduced- or full-dose methylprednisolone (44) showed a non-significant change in CAS. All studies looking at tocilizumab showed a significant improvement in CAS score.

**Table 4:** - CAS.

Reference	Treatment	Mean CAS Before Treatment (SD)	Follow Up (weeks)	Mean CAS After Treatment (SD)	P-value
Dickinson et al., 2004 (24)	Octreotide (LAR)	5.39 (1.56)	24	17/23^d^	<0.001
	Placebo	5.85 (1.51)		22/27^d^	
Stan et al., 2006 (26)	Octreotide (LAR)	6^b^	16	12/14^d^	<0.001
	Placebo	5^b^		4/11^d^	
Salvi et al., 2008 (27)	RTX	4.70 (0.50)	104	1.80 (0.80)	<0.0001
Khanna et al., 2010 (28)	RTX	5.30 (1.0)	8	1.30 (0.50)	0.0001
Silkiss et al., 2010 (29)	RTX	5.5 (1.2)	4, 8, 16, 24, 36, 52	2.3-4.7^a^	<0.01
Mitchell et al., 2013 (31)	RTX	6 (1–8) ^b^	12	2 (1-6)^b^	0.018
Perez-Moreiras et al., 2014 (32)	Tocilizumab	6.5 (1.29)	54	0.61 (0.85)	<0.001
Stan et al., 2015 (33)	Rituximab	4.9 (1)	24, 52	2.0 (1.7)	0.64
	Placebo	5.1(1)		2.9 (2.3)	
Smith et al., 2017 (34)	Teprotumumab	5.1 (0.97)	48	-3.43^a^	<0.001
	Placebo	5.2 (0.74)		-1.85^a^	
Perez-Moreiras et al., 2018 (35)	Tocilizumab	5.0	16, 40	13/15^e^	–
	Placebo	5.0		10/17^e^	
Deltour et al., 2018 (36)	RTX	3.29 (1.16)	24	1.59 (1.12)	<0.001
Insull et al., 2019 (37)	RTX	5.08 (1.98)	27.4	1.58 (1.16)	<0.001
Douglas et al., 2020 (38)	Teprotumumab	5.1 (0.9)	24	24/41^c^	0.001
	Placebo	5.3 (1.0)		9/42^c^	
Ceballos-Macias et al., 2020 (39)	Tocilizumab	4.1 (0.3)	24	1.1 (0.6)	0.001
Perez-Moreiras et al., 2021 (40)	Tocilizumab	6.7 (1.5)	20	0.4 (0.7)	<0.001
Smith et al., 2021 (41)	Tocilizumab	6.78 (1.09)	104	0.44 (0.53)	<0.001
Moi et al., 2021 (42)	Tocilizumab	4.8 (1.13)	24	0.7 (0.82)	–
Ugradar et al., 2022 (43)	Teprotumumab	2.3 (0.9)	24	0.5 (0.7)	0.01
Douglas et al., 2022 (45)	Teprotumumab	3.5 (1.6)	24	4/7^c^	–
	Placebo	3.6 (1.7)	24	21/32^c^	
Bennedjai et al., 2022 (46)	Tocilizumab	5.0 (0.5)	44	1.2 (0.9)	<0.05
	Rituximab	4.0 (1.2)	64	2.5 (1.9)	0.07
Pan et al., 2022 (47)	Doxycycline	–	4	42/48^c^	0.14
			12	42/48^c^	
	Placebo	–	4	38/50^c^	0.82
			12	43/50^c^	
Shen et al., 2022 (48)	MP	4.5 (3-5)^b^	12	2 (1-4)^b^	0.1
	Reduced MP + MTX	4 (3-5)^b^	12	2 (1-3)^b^	0.1
	MP + MTX	4 (4-5)^b^	12	2 (1-3)^b^	0.1
Boutzios et al., 2023 (50)	Tocilizumab	–	30	6/6^d^	0.002
Wang et al., 2023 (51)	Rituximab	4.86 (0.69)	52	0.86 (0.90)	0.001
Men et al., 2024 (52)	Teprotumumab	4.4	24	-3.8 (1.6)^a^	0.01
Hoang et al., 2024 (53)	Teprotumumab	4 (2.5-5)^b^	24	1 (0-3)^b^	0.0002
Rosenblatt et al., 2024 (54)	Teprotumumab	5.11	42	0.84	0.001
Matoc et al., 2024 (55)	Doxycycline	34 (82.9)	12	6 (14.6)	–
	No treatment	19 (46.3)		9 (22)	
Habroosh et al., 2024 (56)	Tocilizumab	7.92 (0.66)	52	2.85 (1.03)	<0.0001
Lee et al., 2024 (57)	Tocilizumab	–	90	16/19^d^	–
Farde et al., 2025 (59)	Tocilizumab	5.22 (1.53)	40	2.09 (1.38)	<0.001
Hiromatsu et al., 2025 (60)	Teprotumumab	4.5 (1.3)	24	16/27^d^	0.0031
	Placebo	4.0 (0.8)		6/27^d^	

**Table 4**. Results of included studies that investigated changes in CAS. CAS: clinical activity score; IV: intravenous; MP: methylprednisolone; MTX: methotrexate; Pred: prednisolone; RTX: rituximab; SD: standard deviation.

a. Mean decrease

b. Median (Range)

c. Number of patients achieving a CAS score of 0 or 1

d. Decrease of 2 or more after treatment

Twenty studies reported proptosis as their main outcome. Nine further studies reported proptosis as a secondary outcome. These results are shown in (**Supplementary Table 6**). Teprotumumab consistently demonstrated the greatest improvement in proptosis, with clinically meaningful reductions across all studies. Similarly, studies looking at tocilizumab also showed consistent and statistically significant reduction. Placebo groups consistently showed little to no change, reinforcing the treatment effect observed with teprotumumab and tocilizumab. The rest of the results were generally mixed, with some studies showing benefit with treatment and others less clear. The quality of data was varied and some studies did not provide data on significance or were statistically insignificant. Studies looking at rituximab yielded variable results, as the improvements were minimal with inconsistent significance. Overall, teprotumumab and tocilizumab appear to be the most effective in reducing proptosis.

Reduction in TRAb levels is documented in (**Supplementary Table 7**). While almost all studies recorded baseline thyroid function tests, only eleven studies measured levels of TRAb in patients. Definite improvements in TRAb levels with tocilizumab use cannot be concluded as there is a statistically significant reduction seen in Ceballos-Macias et al. (36) and Perez-Moreiras et al. (40), but not in Moi et al. (38) and Farde et al. (53). With the exception of Hoang et al. (47) and Deltour et al. (36), all other study results showed no significant change in TRAb associated with treatment.

B Cell depletion is documented in (**Supplementary Table 8**). In total, five studies investigated the immunomodulatory effects of rituximab (27, 29, 31, 37, 51). All studies showed that rituximab treatment was associated with a reduction in B lymphocyte count.

There were seven studies that reported QoL, in all cases as a secondary outcome and these are presented in (**Supplementary Table 9**) (35, 45, 47–49, 52, 60). With the exception of Insull et al. (34), who developed their own questionnaire for the purposes of their trial, all of these studies reported QoL with the Graves’ Ophthalmopathy Quality of Life (GO-QOL) questionnaire, which indicates greater QoL as the total score increases (54). Adverse events from the studies are listed in (**Supplementary Table 10****).**

## Discussion

Overall, this review exhibits a benefit in the use of immunomodulators for thyroid eye disease. While this was seen across the majority of studies included in this review, results were limited by small sample sizes, heterogeneity in outcomes, variability in follow up times and variations in drug comparison.

### Rituximab

Although studies on rituximab showed reductions in CAS, proptosis, and B cell levels, the broader evidence base remains mixed. Two key RCTs assessed rituximab in patients with active, moderate-to-severe GO: one against IV methylprednisolone (61) and one against placebo (33). Salvi et al. (61) found significantly greater CAS improvements in the rituximab arm, with 100% of patients showing improvement at 24 weeks compared to 69% in the steroid arm (p<0.001). In contrast, Stan et al. (33) reported no difference between rituximab and placebo (p=0.75). The discrepancy may be due to differences in disease duration as the patient cohort in the Salvi et al. (61) trial had a shorter average duration of GO. Overall, these findings suggest rituximab could be effective for active GO, however the evidence is limited and further head to head studies are required to compare rituximab against newer immunomodulators such as tocilizumab and teprotumumab.

Furthermore, its use is recommended only in specialized centres due to potential serious adverse events (AEs). Dysthyroid optic neuropathy and severe infusion reactions were reported in the RCTs (33, 61). One study (28) reported a cardiac arrest following a second infusion, resulting in patient death. While causality is unclear, rituximab has been associated with cardiac events such as hypotension, arrhythmia, and cardiogenic shock. A meta-analysis by Shen et al. found inconsistent rates of serious AEs (62). These findings support the cautionary stance in current guidelines (4).

### Tocilizumab

Tocilizumab is shown to effectively reduce CAS and proptosis in multiple observational studies. In Europe, Tocilizumab has been licensed for the treatment of severe rheumatoid arthritis, systemic juvenile idiopathic arthritis, juvenile polyarthritis, giant cell arthritis, cytokine release syndrome and even COVID-19 but not yet for managing GO (63). The study by Perez-Moreiras et al. (35) remains the only RCT looking at tocilizumab in the context of treating GO. The trial provided encouraging evidence for the use of tocilizumab but there were several limitations that could have impacted the overall outcomes. Firstly, the sample size was relatively small, reducing statistical power and leading to potential imbalance between study groups. For example, the baseline GO-QoL score in the tocilizumab group was considerably higher (median 72.2) compared with the placebo group (median 25.0), which may have allowed greater scope for improvement in the latter. Furthermore, there was no limit to disease duration in the inclusion criteria leading to heterogeneity in disease chronicity. This raises the possibility that some participants were not in the active phase of GO at the time of the trial. These factors lead to uncertainty when directly comparing the two cohorts and may have affected the magnitude of treatment response. Nonetheless, despite these methodological limitations, the evidence from the RCT suggests that tocilizumab has positive therapeutic effects, even among patients with long standing disease.

Generally, the safety profile for tocilizumab is favourable, with most patients experiencing mild to moderate side effects. Neutropenia is a recognised side effect of tocilizumab however the studies observed that this was usually transient and resolved with the discontinuation of therapy. Moi et al. (42) reported three cases of malignancy diagnosed after treatment with tocilizumab. Causality cannot be confirmed given the observational nature of the findings and tocilizumab is not known to be associated with an increased risk of cancer. Instead, the observed association may reflect the underlying predisposition linked to autoimmune thyroid disease which has been associated with a higher incidence of certain malignancies – most commonly thyroid, breast, head and neck (64).

### Teprotumumab

Evidence from included studies suggests that teprotumumab is a promising therapy for the treatment of active GO with mild side effects. Smith et al. (34) and Douglas et al. (38) conducted RCTs with comparable designs and sample sizes, both of which found teprotumumab superior to placebo in improving CAS, proptosis, and QoL. Patients with the highest initial proptosis levels appeared to have the largest reductions with teprotumumab, highlighting its efficacy in moderate to severe disease. Patients also experienced rapid therapeutic effects; in Smith et al. 43% achieved a meaningful proptosis response in 6 weeks and Douglas et al. reported a median time to response of 6.4 weeks. In these studies, data was limited on the durability of efficacy in patients with longstanding TED. A follow up study by Douglas et al. (45) looked at the effect on patients with long duration and low disease activity which again demonstrated good treatment effect, highlighting its efficacy regardless of disease severity and duration. However, the open-label design and high loss to follow-up introduces potential bias, highlighting the need for further RCTs to confirm these findings.

Adverse events (AEs) were mostly mild; each RCT reported two serious AEs, with some deemed potentially treatment related. A major recognised side effect of teprotumumab is hearing loss and this was experienced by patients across several studies. Centres delivering teprotumumab should consider regular audiometry and protocols to manage hearing loss associated with its use (65). Overall, current evidence supports the efficacy and general safety of teprotumumab for active GO. However, due to the paucity of data; further research is warranted, in particular long-term and comparative studies. EUGOGO guidelines recommend teprotumumab as a second-line treatment option (4).

### DMARDs

Among the included studies, cyclosporine and methotrexate were evaluated as steroid-sparing agents. Cyclosporine was primarily studied in combination with corticosteroids, limiting the ability to assess its independent efficacy. Meyer et al. reported full visual recovery in patients with dysthyroid optic neuropathy treated with IV methylprednisolone, oral prednisolone, and cyclosporine, but did not report outcomes relevant to this review, such as CAS, proptosis, or QoL (25). Utech et al. (21) found proptosis normalized in 20 of 39 patients treated with cyclosporine and prednisolone. A previous RCT showed cyclosporine alone was less effective than prednisolone (22% vs. 61% response rate; p=0.018), although combination therapy improved outcomes in treatment-resistant patients (66).

Methotrexate was studied in two trials. Shen et al. (48) found no significant differences in CAS, proptosis, or QoL across treatment groups, but reported fewer adverse events in the methotrexate plus reduced-dose methylprednisolone group (p=0.017). Another study of 24 patients receiving methotrexate and IV methylprednisolone showed significant improvements in VISA (an alternative scoring system to CAS) at both intermediate and long-term follow-up, with no serious adverse events reported (67). However, the concurrent use of adjunctive treatments (e.g. cyclosporine, azathioprine, rituximab) are confounding factors and limit the reliability of these findings.

Overall, the evidence supports the potential role of steroid-sparing agents, particularly in combination with corticosteroids. This aligns with EUGOGO guidelines, which recommend IV methylprednisolone in combination with agents like cyclosporine or orbital radiotherapy to enhance steroid efficacy (4). Cyclosporine in combination with oral steroids is considered a valid second-line option, with azathioprine and mycophenolate also noted. Methotrexate, however, is not currently endorsed, likely due to limited supporting data.

### IV Immunoglobulins

IVIG was assessed in two studies, both showing some benefit in reducing proptosis, with Kahaly et al. (22) also noting a reduction in TRAb levels (20, 22). Neither study reported serious adverse events, though IVIG carries a small potential risk of transmitting viral infections, such as HIV and hepatitis B, as it is a blood product (68). Additionally, the need for intravenous administration and associated high costs, limit its practical use. As such, IVIG is not currently considered a viable treatment option for GO (4).

### Other drugs

This review also examined two drugs not routinely used for GO: octreotide and doxycycline. While Ozata et al. (23) reported a reduction in proptosis with octreotide, subsequent studies have shown limited benefit. Three double-blind RCTs on long-acting release octreotide (octreotide-LAR) failed to demonstrate significant improvements in disease outcomes. Dickinson et al. (24) found no benefit in moderately severe GO. Stan et al. reported improvement in CAS but was noted to have overrepresentation of patients with higher baseline CAS in the octreotide-LAR group as well as a small control group (33). The third trial noted a reduction in proptosis but no effect on disease activity in mild cases (69). These RCTs suggest octreotide is not a suitable immunomodulator for GO. This is the position of the EUGOGO guidelines (4), which cite these RCTs as evidence. The guidelines also cite evidence from an RCT on lanreotide, showing no significant differences in CAS or proptosis compared to placebo (70).

Regarding doxycycline, limited evidence is available on its efficacy as an immunomodulator in GO; there is also no advice in the EUGOGO guidelines (4). A 2015 case series reported improvements in CAS and soft tissue swelling in 8/13 patients after treatment with doxycycline (71). However, the small sample size and lack of control group limit the generalizability. Another study in the same year showed improvements in CAS and proptosis with a combined regimen of doxycycline and steroids, though the efficacy of doxycycline alone remains unclear (72). Matoc et al. (55) conducted an RCT that demonstrated significant improvements in quality of life (QOL) and CAS. Although the study reported low rates of adverse events, these findings should be interpreted with caution due to the short duration of the trial and the small sample size. Additionally, the lack of a placebo group, with no treatment as the comparator, limits the ability to draw definitive conclusions about the true efficacy of the intervention.

### Limitations

Due to the heterogeneity of the studies in this review, conducting a meta-analysis was not feasible. This was particularly evident in the reported outcomes. Not every study reported the primary outcome of CAS and there was significant variation in secondary outcomes across all studies. Direct comparison between studies was also limited by a lack of consistency in the reporting of outcomes. Some utilized mean, others median and some even categorical. Furthermore, all the studies consisted of a small number of participants, with 119 patients being the highest patient cohort in a study, limiting the extent to which their results can be extrapolated. The review was also limited to English language studies, which could have excluded other relevant research and introduced bias.

The risk of bias assessments revealed several concerns, particularly in non-randomized trials. These included confounding bias (Domain 1), bias due to missing data from loss to follow-up (Domain 2), outcome measurement bias (Domain 6), and selective reporting (Domain 7). Confounding bias was especially prevalent, often stemming from study designs that allowed prior or concurrent treatments without proper adjustment. For example, Insull et al. (37) investigated rituximab but IV methylprednisolone and optional methotrexate was also concurrently used, without accounting for these confounders.

In contrast, RCTs generally showed a lower risk of bias, though most concerns were highlighted in the randomization process (Domain 1) and bias in the selection of reported results (Domain 5). Overall, this systematic review highlights serious risk of bias in the current literature, especially in non-randomized trials. Future non-randomized studies should adhere more closely to Cochrane ROBINS-I guidelines, particularly by applying statistical methods—such as stratification, regression, matching, standardization, or inverse probability weighting—to control for confounding. Notably, these methods were rarely, if ever, used in the studies reviewed.

### Implications for practice and policy

Overall, this systematic review suggests that tocilizumab and teprotumumab are effective in treating Graves’ orbitopathy (GO), particularly in reducing CAS and proptosis. Combination therapy with steroids and steroid-sparing agents also show benefit. This is seen in Meyer et al. (25) and Shen et al. (48), where both treatments reduced proptosis, with the latter also reducing CAS and TRAb levels. However, based on the data alone, determining the optimal treatment for clinical practice remains challenging. There is a lack of statistical analyses across the studies and variability in methodology such as dosing and outcome measurements prevents direct comparisons between newer and older treatment methods. Dosage and frequency varied even within observational studies looking at a single drug. Heterogeneity of results and methodologies prevent meaningful comparisons of interventions. It highlights the need to standardize outcome measurements for future research. Establishing clinically significant thresholds for improvements in CAS and proptosis would be beneficial in facilitating more reliable comparisons and to establish the true efficacy of emerging therapies.

Additionally, factors such as cost, availability, and patient preferences play a significant role in treatment decisions. While biologics like tocilizumab and teprotumumab may offer greater efficacy and fewer adverse events than established treatments, their high costs limit their widespread use. One treatment course of Teprotumumab costs approximately 360,000 US dollars (73). This is perhaps the reason as to why Teprotumumab is still not widely available in most countries and has only recently been approved in Europe (74). Given this statistic, it is unlikely to be financially viable for public health systems to afford such agents as first line when treatments like steroids are cost-effective and widely accessible. This is also compounded by the fact that other agents, such as rituximab and tocilizumab, may offer similar efficacy in treating active GO and are in comparison less expensive. Furthermore, tocilizumab is available as a subcutaneous preparation, allowing patients to self- administer the treatment at home without having to attend a specialist unit. This could reduce the overall cost burden, as the IV preparation is three times more expensive than the subcutaneous form (75). However, this has yet to be explored in RCTs for GO and could represent a promising avenue for further research to evaluate its efficacy and cost-effectiveness. The EUGOGO guidelines also note the need for further data on teprotumumab’s affordability before it can be considered for first-line use (4). This demonstrates the importance of early referral to specialist centers (e.g. joint thyroid-eye clinics), where a multidisciplinary approach can tailor treatment options to improve patient outcomes.

### Implications for research

While this systematic review has identified a substantial body of research on GO treatment, there are several ways in which this can be improved and built upon. This review has highlighted the need for higher quality primary studies (i.e. RCTs) with large sample sizes, as the majority of studies were small and non-randomized.

Several studies in this review included IV methylprednisolone as part of their treatment regimens, however none compared it to biologics or steroid-sparing agents directly. Similarly, there is a paucity of data on methotrexate as a treatment for GO compared to other steroid-sparing agents. Given the evidence that combination therapy with steroids is more efficacious than steroid-sparing drugs in isolation, there is arguably scope for further RCTs investigating the combined efficacy of methotrexate with steroids, as done by Shen et al. (48).

Emerging treatments also merit further investigation. Long-term RCTs comparing teprotumumab with IV steroids would be beneficial in establishing its safety, sustained efficacy and cost effectiveness. Although teprotumumab is now approved, it is only recommended as a second-line option for active, moderate-to-severe GO. Relatively few patients meet the eligibility criteria for biologic therapies, and their high costs limit their use as first-line treatments within public health systems. Consequently, conducting large-scale RCTs for agents such as teprotumumab can be challenging in some settings. Further research in this area may become possible as teprotumumab becomes more widely accessible.

Similarly, doxycycline requires further evaluation beyond short-term outcomes; additional RCTs should also assess its use in both mild and moderate-to-severe GO. If proven to be beneficial, this could change the outlook of GO treatment given its low cost and accessibility. Novel therapies currently in preclinical development, such as small molecule TSHR antagonists, may prove to be an interesting focus for future clinical trials (76).

## Conclusion

This systematic review highlights positive outcomes for biologics such as tocilizumab and teprotumumab in reducing CAS and proptosis. However, the evidence is limited by heterogeneity between studies and a lack of direct comparisons with standard treatments, hence this review is not conclusive. Future research in the form of RCTs with head-to-head comparisons between drugs are required in order to influence clinical decision-making. Additional research into emerging therapies such as TSH receptor antagonists, constitutes an important direction for future research.

## Data Availability

The original contributions presented in the study are included in the article/supplementary material. Further inquiries can be directed to the corresponding author.

## References

[1] Bartalena L Fatourechi V . Extrathyroidal manifestations of Graves’ disease: a 2014 update. J Endocrinol Invest. (2014) 37:691–700. doi: 10.1007/s40618-014-0097-2, PMID: 24913238

[2] Cockerham KP Chan SS . Thyroid eye disease. Neurol Clin. (2010) 28:3. doi: 10.1016/j.ncl.2010.03.010, PMID: 20637998

[3] Perros P Hegedüs L Bartalena L Marcocci C Kahaly GJ Baldeschi L . Graves’ orbitopathy as a rare disease in Europe: a European Group on Graves’ Orbitopathy (EUGOGO) position statement. Orphanet J Rare Dis. (2017) 12:1. doi: 10.1186/s13023-017-0625-1, PMID: 28427469 PMC5397790

[4] Bartalena L Kahaly GJ Baldeschi L Dayan CM Eckstein A Marcocci C . The 2021 European Group on Graves’ orbitopathy (EUGOGO) clinical practice guidelines for the medical management of Graves’ orbitopathy. Eur J Endocrinol. (2021) 185:4. doi: 10.1530/EJE-21-0479, PMID: 34297684

[5] Cawood T Moriarty P O’Shea D . Recent developments in thyroid eye disease. BMJ. (2004) 329:7462. doi: 10.1136/bmj.329.7462.385, PMID: 15310608 PMC509348

[6] Eckstein AK Plicht M Lax H Neuhäuser M Mann K Lederbogen S . Thyrotropin receptor autoantibodies are independent risk factors for graves’ Ophthalmopathy and help to predict severity and outcome of the disease. J Clin Endocrinol Metab. (2006) 91:9. doi: 10.1210/jc.2005-2813, PMID: 16835285

[7] Diana T Ponto KA Kahaly GJ . Thyrotropin receptor antibodies and Graves’ orbitopathy. J Endocrinol Invest. (2021) 44:4. doi: 10.1007/s40618-020-01380-9, PMID: 32749654 PMC8310479

[8] Gillespie EF Smith TJ Douglas RS . Thyroid eye disease: towards an evidence base for treatment in the 21st century. Curr Neurol Neurosci Rep. (2012) 12:3. doi: 10.1007/s11910-012-0256-9, PMID: 22354545 PMC3463137

[9] Mourits MPH Prummel MF Wiersinga WM Koornneef L . Clinical activity score as a guide in the management of patients with Graves’ ophthalmopathy. Clin Endocrinol (Oxf). (1997) 47:1. doi: 10.1046/j.1365-2265.1997.2331047.x, PMID: 9302365

[10] Mourits MP Koornneef L Wiersinga WM Prummel MF Berghout A van der Gaag R . Clinical criteria for the assessment of disease activity in Graves' ophthalmopathy: a novel approach. Br J Ophthalmol. (1989) 73:639–44. doi: 10.1136/bjo.73.8.639, PMID: 2765444 PMC1041835

[11] Zang S Ponto KA Kahaly GJ . Intravenous glucocorticoids for graves’ Orbitopathy: efficacy and morbidity. J Clin Endocrinol Metab. (2011) 96:2. doi: 10.1210/jc.2010-1962, PMID: 21239515

[12] Zoumalan CI Cockerham KP Turbin RE Volpe NJ Kazim M Douglas RS . Efficacy of corticosteroids and external beam radiation in the management of moderate to severe thyroid eye disease. J Neuro-Ophthalmol. (2007) 27:3. doi: 10.1097/WNO.0b013e31814a5ef8, PMID: 17895822

[13] Bhatti MT Dutton JJ . Thyroid eye disease. J Neuro-Ophthalmol. (2014) 34:2. doi: 10.1097/WNO.0000000000000128, PMID: 24821102

[14] Men CJ Kossler AL Wester ST . Updates on the understanding and management of thyroid eye disease. Ther Adv Ophthalmol. (2021) 13. doi: 10.1177/25158414211027760, PMID: 34263138 PMC8252358

[15] Food and Drug Administration . FDA approves first treatment for thyroid eye disease (2020). Available online at: https://www.fda.gov/news-events/press-announcements/fda-approves-first-treatment-thyroid-eye-disease (Accessed April 17, 2023).

[16] Page MJ McKenzie JE Bossuyt PM Boutron I Hoffmann TC Mulrow CD . The PRISMA 2020 statement: an updated guideline for reporting systematic reviews. BMJ. (2021) 372:n71. doi: 10.1136/bmj.n71, PMID: 33782057 PMC8005924

[17] Sterne JAC Savović J Page MJ Elbers RG Blencowe NS Boutron I . RoB 2: a revised tool for assessing risk of bias in randomised trials. BMJ. (2019) 366:l4898. doi: 10.1136/bmj.l4898, PMID: 31462531

[18] Sterne JAC Hernán MA Reeves BC Savović J Berkman ND Viswanathan M . ROBINS-I: a tool for assessing risk of bias in non-randomised studies of interventions. BMJ. (2016) 355:i4919. doi: 10.1136/bmj.i4919, PMID: 27733354 PMC5062054

[19] McGuinness LA Higgins JPT . Risk-of-bias VISualization (robvis): An R package and Shiny web app for visualizing risk-of-bias assessments. Res Synth Methods. (2020) 12:1. doi: 10.1002/jrsm.1411, PMID: 32336025

[20] Antonelli A Saracino A Alberti B Canapicchi R Cartei F Lepri A . High-dose intravenous immunoglobulin treatment in Graves’ ophthalmopathy. Acta Endocrinol (Copenh). (1992) 126:1. doi: 10.1530/acta.0.1260013, PMID: 1736548

[21] Utech CI Khatibnia U Winter PF Wulle KG . MR T2 relaxation time for the assessment of retrobulbar inflammation in graves’ Ophthalmopathy. Thyroid. (1995) 5:3. doi: 10.1089/thy.1995.5.185, PMID: 7580266

[22] Kahaly G Pitz S Müller-Forell W Hommel G . Randomized trial of intravenous immunoglobulins versus prednisolone in Graves’ ophthalmopathy. Clin Exp Immunol. (1996) 106:2. doi: 10.1046/j.1365-2249.1996.d01-854.x, PMID: 8918563 PMC2200583

[23] Ozata M Bolu E Sengul A Tasar M Beyhan Z Corakci A . Effects of octreotide treatment on graves’ Ophthalmopathy and circulating sICAM-1 levels. Thyroid. (1996) 6:4. doi: 10.1089/thy.1996.6.283, PMID: 8875747

[24] Dickinson A Vaidya B Miller M Coulthard A Perros P Baister E . Double-blind, placebo-controlled trial of octreotide long-acting repeatable (LAR) in thyroid-associated ophthalmopathy. J Clin Endocrinol Metab. (2004) 89:5910–5. doi: 10.1210/jc.2004-0697, PMID: 15579735

[25] Meyer PAR . Avoiding surgery for thyroid eye disease. Eye. (2006) 20:10. doi: 10.1038/sj.eye.6702393, PMID: 17019415

[26] Stan MN Garrity JA Bradley EA Woog JJ Bahn MM Brennan MD . Randomized, double-blind, placebo-controlled trial of long-acting release octreotide for treatment of graves’ Ophthalmopathy. J Clin Endocrinol Metab. (2006) 91:4817–24. doi: 10.1210/jc.2006-1105, PMID: 16984988

[27] Salvi M Vannucchi G Campi I Currò N Beck-Peccoz P . New immunomodulators in the treatment of Graves’ ophthalmopathy. Ann Endocrinol (Paris). (2008) 69:2. doi: 10.1016/j.ando.2008.02.013, PMID: 18417090

[28] Khanna D Chong KKL Afifiyan NF Hwang CJ Lee DK Garneau HC . Rituximab treatment of patients with severe, corticosteroid-resistant thyroid-associated ophthalmopathy. Ophthalmology. (2010) 117:1. doi: 10.1016/j.ophtha.2009.05.029, PMID: 19818507 PMC2814962

[29] Silkiss RZ Reier A Coleman M Lauer SA . Rituximab for thyroid eye disease. Ophthalmic Plast Reconstr Surg. (2010) 26:5. doi: 10.1097/IOP.0b013e3181c4dfde, PMID: 20562667

[30] Tambe K Bhargava J Tripathi A Gregory M Burns J Sampath R . The role of intravenous methylprednisolone immunosuppression in the management of active thyroid eye disease. Orbit. (2010) 29:5. doi: 10.3109/01676831003660663, PMID: 20812826

[31] Mitchell AL Gan EH Morris M Johnson K Neoh C Dickinson AJ . The effect of B cell depletion therapy on anti-TSH receptor antibodies and clinical outcome in glucocorticoid-refractory Graves’ orbitopathy. Clin Endocrinol (Oxf). (2013) 79:3. doi: 10.1111/cen.12141, PMID: 23320840

[32] Pérez-Moreiras JV Álvarez-López A Gómez EC . Treatment of active corticosteroid-resistant graves’ Orbitopathy. Ophthalmic Plast Reconstr Surg. (2014) 30:162–7. doi: 10.1097/IOP.0000000000000037, PMID: 24503568

[33] Stan MN Garrity JA Carranza BG Prabin T Bradley EH Bahn RS . Randomized controlled trial of rituximab in patients with graves’ Orbitopathy. J Clin Endocrinol Metab. (2015) 100:432–41. doi: 10.1210/jc.2014-2572, PMID: 25343233 PMC4318907

[34] Smith TJ Kahaly GJ Ezra DG Fleming JC Dailey RA Tang RA . Teprotumumab for thyroid-associated ophthalmopathy. New Engl J Med. (2017) 376:1748–61. doi: 10.1056/NEJMoa1614949, PMID: 28467880 PMC5718164

[35] Perez-Moreiras JV Gomez-Reino JJ Maneiro JR Perez-Pampin E Romo Lopez A Rodríguez Alvarez FM . Efficacy of tocilizumab in patients with moderate-to-severe corticosteroid-resistant graves orbitopathy: A randomized clinical trial. Am J Ophthalmol. (2018) 195:181–90. doi: 10.1016/j.ajo.2018.07.038, PMID: 30081019

[36] Deltour JB D’Assigny FM Ladsous M Giovansili L Cariou B Caron P . Efficacy of rituximab in patients with Graves’ orbitopathy: a retrospective multicenter nationwide study. Graefe s Arch Clin Exp Ophthalmol. (2020) 258:2013–21. doi: 10.1007/s00417-020-04651-6, PMID: 32405700

[37] Insull EA Sipkova Z David J Turner HE Norris JH . Early low-dose rituximab for active thyroid eye disease: An effective and well-tolerated treatment. Clin Endocrinol (Oxf). (2019) 91:1. doi: 10.1111/cen.13970, PMID: 30864162

[38] Douglas RS Kahaly GJ Patel A Sile S Thompson EHZ Perdok R . Teprotumumab for the treatment of active thyroid eye disease. New Engl J Med. (2020) 382:341–52. doi: 10.1056/NEJMoa1910434, PMID: 31971679

[39] Ceballos-Macías José J Rivera-Moscoso R Flores-Real Jorge A Vargas-Sánchez J Ortega-Gutiérrez G Madriz-Prado R . Tocilizumab in glucocorticoid-resistant graves orbitopathy. A case series report of a Mexican population. Annales d’Endocrinolo. (2020) 81:78–82. doi: 10.1016/j.ando.2020.01.003, PMID: 32340849

[40] Pérez-Moreiras JV Varela-Agra M Prada-Sánchez MC Prada-Ramallal G . Steroid-resistant graves’ Orbitopathy treated with tocilizumab in real-world clinical practice: A 9-year single-center experience. J Clin Med. (2021) 10:706. doi: 10.3390/jcm10040706, PMID: 33670151 PMC7916878

[41] Smith LD Moscato EE Seiff SR . Tocilizumab for the management of thyroid-associated orbitopathy. Ophthalmic Plast Reconstr Surg. (2021) 38:188–92. doi: 10.1097/IOP.0000000000002027, PMID: 34293786

[42] Moi L Hamedani M Ribi C . Long-term outcomes in corticosteroid-refractory Graves’ orbitopathy treated with tocilizumab. Clin Endocrinol. (2021) 97:363–70. doi: 10.1111/cen.14655, PMID: 34908176 PMC9545295

[43] Ugradar S Kang J Kossler AL Zimmerman E Braun J Harrison AR . Teprotumumab for the treatment of chronic thyroid eye disease. Eye. (2021) 36:1553–9. doi: 10.1038/s41433-021-01593-z, PMID: 34244669 PMC9307784

[44] Ozzello DJ Dallalzadeh LO Liu CY . Teprotumumab for chronic thyroid eye disease. Orbit. (2021) 41:539–46. doi: 10.1080/01676830.2021.1933081, PMID: 34060414

[45] Douglas RS Kahaly GJ Ugradar S Elflein H Ponto KA Fowler BT . Teprotumumab efficacy, safety, and durability in longer-duration thyroid eye disease and re-treatment. Ophthalmology. (2022) 129:4. doi: 10.1016/j.ophtha.2021.10.017, PMID: 34688699

[46] Bennedjaï A Bouheraoua N Gatfossé M Dupasquier-Fediaevsky L Errera MH Tazartes M . Tocilizumab versus rituximab in patients with moderate to severe steroid-resistant graves’ Orbitopathy. Ocular Immunol Inflammat. (2020) 30:500–5. doi: 10.1080/09273948.2020.1808688, PMID: 32965148

[47] Pan Y Chen YX Zhang J Lin ML Liu GM Xu XL . Doxycycline vs Placebo at 12 Weeks in Patients With Mild Thyroid-Associated Ophthalmopathy. JAMA Ophthalmol. (2022) 140:11. doi: 10.1001/jamaophthalmol.2022.3779, PMID: 36173609 PMC9523551

[48] Shen L Ye L Zhu W Jiao Q Zhou Y Wang S . Methotrexate plus reduced or full-dose glucocorticoids for the treatment of active, moderate-to-severe Graves’ orbitopathy. Eur Thyroid J. (2022) 11:5. doi: 10.1530/ETJ-22-0017, PMID: 35900774 PMC9422237

[49] Douglas RS Couch SM Wester ST Fowler B Liu CY Subramanian PS . Efficacy and safety of teprotumumab in thyroid eye disease patients with long duration and low disease activity. J Clin Endocrinol Metab. (2023) 109:25–35. doi: 10.1210/clinem/dgad637, PMID: 37925673 PMC10735297

[50] Boutzios G Chatzi S Goules AV Mina A Charonis GC Vlachoyiannopoulos PG . Tocilizumab improves clinical outcome in patients with active corticosteroid-resistant moderate-to-severe Graves’ orbitopathy: an observational study. Front Endocrinol. (2023) 14. doi: 10.3389/fendo.2023.1186105, PMID: 37424868 PMC10327634

[51] Wang Y Hu H Chen L Zhang H Yang T Xu X . Observation study of using a small dose of rituximab treatment for thyroid-associated ophthalmopathy in seven Chinese patients: One pilot study. Front Endocrinol. (2023) 13. doi: 10.3389/fendo.2022.1079852, PMID: 36743915 PMC9889535

[52] Men CJ Amarikwa L Pham B Sears C Clauss K Lee BW . Teprotumumab for the treatment of recalcitrant thyroid eye disease. Ophthalmic Plast Reconstr Surg. (2024) 40:276. Available online at: https://journals.lww.com/op-rs/abstract/2024/05000/teprotumumab_for_the_treatment_of_recalcitrant.7.aspx (Accessed October 20, 2025). 37972960 10.1097/IOP.0000000000002564 PMC11090759

[53] Hoang TD Flor RJ de la Torre S Nguyen C Raiciulescu S Shakir MKM . Effects of teprotumumab and role of human leukocyte antigens markers in patients with thyroid eye disease. Endocr Pract. (2024) 30:11. doi: 10.1016/j.eprac.2024.08.005, PMID: 39187158

[54] Rosenblatt TR Chiou CA Yoon MK Wolkow N Lee NG Freitag SK . Proptosis regression after teprotumumab treatment for thyroid eye disease. Ophthalmic Plast Reconstr Surg. (2023) 40:187–91. doi: 10.1097/IOP.0000000000002531, PMID: 37791840

[55] Matoc I Kasa K Kasumovic A Prpić A Vukojević A Zrinščak O . One incremental stride for doxycycline, one substantial advancement for thyroid eye disease. Diagnos (Basel). (2024) 14:791. doi: 10.3390/diagnostics14080791, PMID: 38667437 PMC11049125

[56] Habroosh FA Albrashdi SS Alsaadi AH Eatamadi H . Tocilizumab use for optic nerve compression in thyroid eye disease: a prospective longitudinal cohort. Int Ophthalmol. (2024) 44. doi: 10.1007/s10792-024-03143-4, PMID: 38717530

[57] Lee C Park JW Kim YD Woo KI . Efficacy of tocilizumab in patients with moderate-to-severe corticosteroid-resistant thyroid eye disease: a prospective study. Int Ophthalmol. (2024) 44. doi: 10.1007/s10792-024-03117-6, PMID: 38622479

[58] Al-Sharif EM Zhou J Shoji MK Acuff K Liu CY Korn BS . Effects of teprotumumab on eyelid retraction in thyroid eye disease. Ophthalmic Plast Reconstr Surg. (2024) 41:22–7. doi: 10.1097/IOP.0000000000002707, PMID: 38722772

[59] Farde K Träisk F . Tocilizumab – a disease-modulating treatment for thyroid associated ophthalmopathy? Orbit. (2025) 44:415–9. doi: 10.1080/01676830.2025.2452181, PMID: 39840782

[60] Hiromatsu Y Ishikawa E Kozaki A Takahashi Y Tanabe M Hayashi K . A randomised, double-masked, placebo-controlled trial evaluating the efficacy and safety of teprotumumab for active thyroid eye disease in Japanese patients. Lancet Regional Health - Western Pac. (2025) 55:101464–4. Available online at: https://www.thelancet.com/journals/lanwpc/article/PIIS2666-6065(25)00001-X/fulltext?uuid=uuid%3Ad9caf0da-20fc-45e6-9949-e42844e2c443 (Accessed October 20, 2025). 10.1016/j.lanwpc.2025.101464 PMC11787687 39896230

[61] Salvi M Vannucchi G Currò N Campi I Covelli D Dazzi D . Efficacy of B-cell targeted therapy with rituximab in patients with active moderate to severe graves’ Orbitopathy: A randomized controlled study. J Clin Endocrinol Metab. (2015) 100:2. doi: 10.1210/jc.2014-3014, PMID: 25494967 PMC4318899

[62] Shen WC Lee CH Loh EW Hsieh AT Chen L Tam KW . Efficacy and safety of rituximab for the treatment of graves’ Orbitopathy: A meta-analysis of randomized controlled trials. Pharmacotherapy. (2018) 38:5. doi: 10.1002/phar.2111, PMID: 29601105

[63] RoActemra . European medicines agency. Amsterdam, The Netherlands: European Medicines Agency (2018). Available online at: https://www.ema.europa.eu/en/medicines/human/EPAR/roactemra (Accessed October 20, 2025).

[64] Shu X Ji J Li X Sundquist J Sundquist K Hemminki K . Cancer risk in patients hospitalized for Graves' disease: a population-based cohort study in Sweden. Br J Can. (2010) 102:1397–9. doi: 10.1038/sj.bjc.6605624, PMID: 20354521 PMC2865750

[65] Keen JA Correa T Pham C Claussen AD Hansen MR Carter KD . Frequency and patterns of hearing dysfunction in patients treated with teprotumumab. Ophthalmology. (2024) 131:30–6. doi: 10.1016/j.ophtha.2023.08.001, PMID: 37567417

[66] Prummel MF Mourits MPH Berghout A Krenning EP van der Gaag R Koornneef L . Prednisone and cyclosporine in the treatment of severe graves’ Ophthalmopathy. N Engl J Med. (1989) 321:20. doi: 10.1056/NEJM198911163212002, PMID: 2519530

[67] Sipkova Z Insull EA David J Turner HE Keren S Norris JH . Early use of steroid-sparing agents in the inactivation of moderate-to-severe active thyroid eye disease: a step-down approach. Clin Endocrinol (Oxf). (2018) 89:6. doi: 10.1111/cen.13834, PMID: 30103255

[68] Chaigne B Mouthon L . Mechanisms of action of intravenous immunoglobulin. Transfus Apher Sci. (2017) 56:1. doi: 10.1016/j.transci.2016.12.017, PMID: 28161150

[69] Wémeau JL Caron P Beckers A Rohmer V Orgiazzi J Borson-Chazot F . Octreotide (Long-acting release formulation) treatment in patients with graves’ Orbitopathy: clinical results of a four-month, randomized, placebo-controlled, double-blind study. J Clin Endocrinol Metab. (2005) 90:2. doi: 10.1210/jc.2004-1334, PMID: 15562016

[70] Chang TC Liao SL . Slow-release lanreotide in Graves’ ophthalmopathy: A double-blind randomized, placebo-controlled clinical trial. J Endocrinol Invest. (2006) 29:5. doi: 10.1007/BF03344124, PMID: 16794364

[71] Lin M Mao Y Ai S Liu G Zhang J Yan J . Efficacy of subantimicrobial dose doxycycline for moderate-to-severe and active graves’ Orbitopathy. Int J Endocrinol. (2015) 2015:285698. doi: 10.1155/2015/285698, PMID: 26221138 PMC4499606

[72] Mendoza-Martínez P Cacho D Sosa-Caballero A Domínguez-Hernández L . Effectiveness of methylprednisolone combined with doxycycline to reduce the clinical activity of Graves orbitopathy. Med Int Méx. (2022) 38:3. doi: 10.24245/mim.v38i3.5764

[73] Martel A Rocher F Gerard A . Teprotumumab for the treatment of thyroid eye disease: why should we keep our eyes “Wide open”?—A clinical and pharmacovigilance point of view. J Pers. Med. (2024) 14:1027. doi: 10.3390/jpm14101027, PMID: 39452535 PMC11508897

[74] Tepezza . European medicines agency (EMA). Amsterdam, The Netherlands: European Medicines Agency (EMA (2025). Available online at: https://www.ema.europa.eu/en/medicines/human/EPAR/tepezza (Accessed October 20, 2025).

[75] Silkiss RZ Paap MK Roelofs KA Agi J . Treatment of corticosteroid resistant thyroid eye disease with subcutaneous tocilizumab. Can J Ophthalmol. (2021) 56:66–70. doi: 10.1016/j.jcjo.2020.07.020, PMID: 32919997

[76] Neumann S . Small molecule agonists and antagonists as potential new therapeutics targeting the TSH receptor. Endocr Abstracts. (2017) 49:74–6. doi: 10.1530/endoabs.49.NSA5.2

