# Supplementary material for: Immunomodulators in Graves’ ophthalmopathy: a systematic review
Source: Front Endocrinol (Lausanne). 2026 Feb 10;17:1686786. doi: 10.3389/fendo.2026.1686786 (PMC12929135; doi:10.3389/fendo.2026.1686786)
Supplement: Supplementary file 1 [file Table1.docx]

***Supplementary Material***

# Supplementary Figures and Tables

**Supplementary Table 1 - Patient Characteristics**

| Study  Reference | Intervention | Average age (years) | Gender (M \| F) | Thyroid disease | Smoking | Previous thyroid treatment | Current thyroid treatment |
| --- | --- | --- | --- | --- | --- | --- | --- |
| Antonelli et al., 1992 (20) | Group 1: IVIG + OR | 46.7 | 1 \| 6 | All GD  12 hyperthyroid  7 euthyroid | - | - | 6 MMI |
|  | Group 2: IVIG | 45.7 | 3 \| 4 |  |  |  | 5 MMI |
|  | Group 3: MP + OR | 48 | 1 \| 11 |  |  |  | 9 MMI |
| Utech et al., 1995 (21) | Cyclosporine | 48 | 9 \| 30 | Intractable, severe GO (Class 2-6 NOSPECS) | - | 39 steroids  9 RR  7 surgical | 39 Thyroxine  (for thyroid ablation) |
| Kahaly et al., 1996 (22) | IVIG | 48 | 5 \| 16 | All GD, euthyroid for >2 months | - | - | MMI 5-20mg/day |
|  | Prednisolone | 47 | 4 \| 15 |  |  |  |  |
| Ozata et al., 1996 (23) | Octreotide | 35.2 | 3 \| 7 | All GD + euthyroid | - | MMI + propranolol | MMI |
| Dickinson et al., 2004 (24) | Octreotide | 50 | 4 \| 19 | Euthyroid  Active GO | 12 | 9 Orbital radiation  16 IVMP | 46/50 treated for hyperthyroidism |
|  | Placebo |  | 7 \| 20 |  | 13 |  |  |
| Meyer et al., 2006 (25) | IVMP | 54 | 2 \| 12 | - | - | - | - |
|  | Pred |  |  |  |  |  |  |
|  | Cyclosporine |  |  |  |  |  |  |
| Stan et al., 2006 (26) | Octreotide | 53 | 3 \| 11 | GD  Active GO  Euthyroid | 4 | 19 RAI  10 IVMP  5 antithyroid drugs | - |
|  | Placebo | 61 | 3 \| 7 |  | 5 |  |  |
| Salvi et al., 2008 (27) | IV RTX | 31-51 | 2 \| 7 | All GD  7 active GO  2 mild lid signs | - | - | MMI |
|  | IVMP | 30-82 | 3 \| 17 | - |  |  |  |
| Khanna et al., 2010 (28) | RTX | 54.3 | 2 \| 4 | All GD + euthyroid | 3 | 2 decompression  2 RAI + thyroxine  4 antithyroid drugs | - |
| Silkiss et al., 2010 (29) | RTX | 52.1 | 5 \| 7 | 10 hyperthyroid  1 subclinical  1 euthyroid | 4 | 3 RAI  2 MMI  1 PTU  1 liothyronine  1 MMI + thyroidectomy |  |
| Tambe et al., 2010 (30) | IVMP | - | - | GD  32 hyperthyroid  2 hypothyroid  2 euthyroid | 20 | - | - |
| Mitchell et al., 2013 (31) | RTX | 62 | 1 \| 8 | GD  7 hyperthyroid  1 hypothyroid  1 euthyroid | - | All IVMP  4 RAI | All levothyroxine  1 PTU  3 carbimazole |
| Perez-Moreiras et al., 2014 (32) | Tocilizumab | 47.9 | 2 \| 16 | Active GO | 9 | All IVMP  3 RT | - |
| Stan et al., 2014 (33) | RTX | 13 | 4 \| 9 | GD  Euthryoid | 2 | - | - |
|  | Placebo | 12 | 4 \| 8 |  | 2 |  |  |
| Smith et al., 2017 (34) | Teprotumumab | 51.6 | 28 \| 15 | GD | 11 | Oral glucocorticoids | 15Antithyroid drug  26 Levothryoxine |
|  | Placebo | 54.2 | 36 \| 8 |  | 18 |  | 20 Antithyroid drug  23 Levothryoxine |
| Perez-Moreiras et al., 2018 (35) | Tocilizumab | 15 | 4 \| 11 | Active GO  Euthyroid | 0 | IVMP | - |
|  | Placebo | 17 | 4 \| 13 |  | 0 |  |  |
| Deltour et al., 2018 (36) | RTX | 51.2 | 27 \| 13 | GO  GD | 25 | IVMP | - |
| Insull et al., 2019 (37) | RTX | 49 | 4 \| 8 | GD  5 hyperthyroid  7 euthyroid | 5 | 4 thyroidectomy  3 oral prednisolone  3 IVMP | 6 carbimazole  4 levothyroxine  2 none |
| Douglas et al., 2020 (38) | Teprotumumab | 51.6 | 12 \| 29 | TED  GD | 9 | - | - |
|  | Placebo | 48.9 | 11 \| 31 |  | 8 |  |  |
| Ceballos-Macias et al., 2020 (39) | Tocilizumab | 45.9 | 6 \| 2 | - | 2 | 6 oral steroids  8 IV steroids  4 MTX  1 Rituxamab  2 RT | - |
| Perez-Moreiras et al. 2021 (40) | Tocilizumab | 53.8 | 13 \| 41 | Active GO  Not responsive to glucocorticoids | 21 | 10 RAI  10 thyroidectomy | - |
| Smith et al. 2021 (41) | Tocilizumab | 55.6 | 0 \| 9 | Active GO | 1 | - | - |
| Moi et al., 2021 (42) | Tocilizumab | 51 | 3 \| 7 | GD | 6 | 3 orbital radiotherapy  1 RAI  10 oral steroids | - |
| Ugradar et al., 2022 (43) | Teprotumumab | 57 | 8 \| 23 | TED | 0 | - | - |
| Ozello et al., 2022 (44) | Teprotumumab | 50.2 | 2 \| 7 | TED | 1 | - | - |
| Douglas et al., 2022 (45) | Teprotumumab | 56.1 | 3 \| 11 | GD | 3 | - | - |
|  | Placebo | 48.5 | 10 \| 27 |  | 8 |  |  |
| Bennedjai et al., 2022 (46) | Tocilizumab | 50 | 4 \| 3 |  | 3 | 2 surgical decompression  10 IVMP  1 oral steroid  2 orbital radiotherapy | - |
|  | Rituximab | 50 | 3 \| 11 |  | 8 | 8 IVMP  12 oral steroid  8 orbital radiotherapy |  |
| Pan et al., 2022 (47) | Doxycycline | 38.1 (9.5) | 9 \| 41 | GD  Autoimmune thyroiditis Nodular goiter | 12 | 6 RAI  4 thyroidectomy  26 medical management | 19 PTU + MMI  3 levothyroxine  2 PTU + MMI + levothyroxine |
|  | Placebo | 35.6 (8.8) | 16 \| 34 |  | 12 | 5 RAI  1 thyroidectomy  27 medical management | 20 PTU + MMI  3 PTU + MMI + levothyroxine |
| Shen et al., 2022 (48) | Group 1: MP | 46.5 (35-52) | 9 \| 30 | 29 GD (1 pt not accounted for)  23 Euthyroid | 0 | 27 antithyroid  6 RAI  1 thyroidectomy | 6 levothyroxine  5 MMI  1 PTU  12 levothyroxine + MMI  4 levothyroxine + PTU |
|  | Group 2: Reduced MP + MTX | 46 (37-53) | 7 \| 23 | All GD  25 Euthyroid | 2 | 28 antithyroid drugs  8 RAI | 2 levothyroxine  5 MMI  10 PTU  12 levothyroxine + PTU |
|  | Group 3: full-dose MP | 46.5 (39-53) | 10 \| 20 | All GD  23 Euthyroid | 0 | 27 antithyroid drugs  5 RAI  1 thyroidectomy | 2 levothyroxine  2 MMI  7 PTU  1 levothyroxine + MMI 14 levothyroxine + PTU |
| Douglas et al., 2023 (49) | Teprotumumab | 48.6 | 10 \| 32 | TED | 6 | - | - |
|  | Placebo | 48.9 | 2 \| 18 |  | 2 |  |  |
| Boutioz et al., 2023 (50) | Tocilizumab | 58.4 | 6 \| 6 | GO not responsive to glucocorticoids | 8 | 2 thyroidectomy  1 ocular surgery | - |
| Wang et al., 2023 (51) | Rituximab | 51.1 | 2 \| 5 | 3 Euthyroid  3 hyperthyroid  1 hypothyroid | 2 | 4 IVMP  1 somatostatin | 2 MMI  2 levothyroxine post RT |
| Men et al., 2023 (52) | Teprotumumab | 59.3 | 20 \| 46 | 58 Active GO  8 Inactive | 6 | 20 radioiodine  13 methimazole / PTU  18 thyroidectomy  20 orbital decompression  19 orbital radiation therapy  6 tocilizumab | - |
| Hoang et al., 2024 (53) | Teprotumumab | 47.6 | 7 \| 19 | 25 GD  1 no history of GD but MRI with TED | 7 | 20 MMI  5 RAI  10 thyroidectomy  2 RAI + thyroidectomy | - |
| Rosenblatt et al., 2024 (54) | Teprotumumab | 56.8 | 29 \| 90 | - | 9 | - | - |
| Matoc et al., 2024 (55) | Doxycycline | 50 | 10 \| 72 | GD  HT  Patients with thyroid antibodies | 28 | - | - |
|  | No treatment | 52 |  |  |  |  |  |
| Habroosh et al., 2024 (56) | Tocilizumab | 49.23 | 6 \| 7 | DON secondary to severe TED | 3 | 4 8 antithyroid drugs  2 IVMP and orbital decompression | - |
| Lee et al., 2024 (57) | Tocilizumab | 46.4 | 6 \| 13 | 3 euthyroid  12 hyperthyroid  4 hypothyroid | 4 | 3 thyroidectomy  13 RT | - |
| Al-Sharif et al., 2024 (58) | Teprotumumab | 52.02 | 12 \| 79 | - | - | - | - |
| Farde et al., 2025 (59) | Tocilizumab | 63.8 | 4 \| 19 | - | 9 | 12 thyroidectomy  4 RAI  7 antithyroid drugs | - |
| Hiromatsu et al., 2025 (60) | Teprotumumab | 46.6 | 9 \| 18 | GD  Active GO | 4 | - | - |
|  | Placebo | 50.0 | 7 \| 20 |  | 4 |  |  |

Supplementary Table 4. Patient characteristics of included studies.

DON: GD: Graves Disease, RR: retrobulbar radiation, RAI: Radioactive iodine therapy, MMI: methimazole, PTU: Propylthiouracil, RT: radiotherapy, HT: Hashimoto’s Thyroiditis.

**Supplementary Table 2 - Proptosis**

| Ref | Drug(s) studied | Mean proptosis in mm before treatment (SD) | Mean proptosis in mm after treatment (SD) | P-value |
| --- | --- | --- | --- | --- |
| Antonelli et al., 1992 (20) | IVIG + OR | 22.4 (2.4) | 20.6 (3.0) | - |
|  | IVIG | 22.6 (3.2) | 20.6 (3.2) |  |
|  | MP + OR | 22.5 (3.2) | 20.1 (2.1) |  |
| Kahaly et al., 1996 (22) | IVIG | 24.5 (22-26) ^a^ | 22 (19-22)^a^ | <0.005 |
|  | Pred | 25 (21-27)^a^ | 22 (19-23)^a^ | <0.005 |
| Ozata et al., 1996  (23) | Octreotide | OD: 21.2 (3.30)  OS: 21.0 (2.90) | OD: 20.8 (2.50)  OS: 20.2 (2.40) | - |
| Dickinson et al., 2004 (24) | Octreotide | 23.09 (2.85) | -^c^ |  |
|  | Placebo | 21.21 (3.39) | -^c^ |  |
| Khanna et al., 2010 (28) | Rituximab | 24 (3.70) | 23.6 (3.70) | 0.17 |
| Tambe et al., 2010 (30) | IVMP | - | 1.31 (0-3) | - |
| Perez-Moreiras et al., 2014 (32) | Tocilizumab | 22.33 (3.16) | −3.92 (1.54)^b^ | - |
| Stan et al., 2015 (33) | Rituximab | OD: 24.6 (3.0) OS: 24.2 (3.3) | OD: -0.82 (1.4)^b^  OS: -0.1 (1.2)^b^ | - |
|  | Placebo | OD: 23.3 (3.8)  OS: 23.0 (2.4) | OD: 0.0^b^  OS:0.0^b^ |  |
| Smith et al., 2017 (34) | Teprotumumab | 23.4 (3.2) | -2.46^b^ | <0.001 |
|  | Placebo | 23.1 (2.9) | -0.15^b^ |  |
| Deltour et al., 2018 (36) | RTX | 21.84 (2.59) | 20.94 (3.59) | 0.11 |
| Douglas et al., 2020 (38) | Teprotumumab | 22.62 (3.32) | -3.32^b^ | - |
|  | Placebo | 23.20 (3.21) | -0.53^b^ |  |
| Ceballos-Macias et al., 2020 (39) | Tocilizumab | 21.2 (3.2) | 19.3 (2.0) | 0.02 |
| Perez-Moreiras et al. 2021 (40) | Tocilizumab | 21.8 | 19.5 | <0.001 |
| Moi et al., 2021 (42) | Tocilizumab | 23.20 (2.10) | 20.60 (2.01) | <0.001 |
| Ugradar et al., 2022 (43) | Teprotumumab | 24.0 (3.8) | 21.0 (SD 4.0) | <0.01 |
| Ozello et al., 2022 (44) | Teprotumumab | - | -4.0 (2.4)^b^ | 0.002 |
| Douglas et al., 2022 (45) | Placebo | 23.7 (2.6) | 23.4 (2.5) | 0.001 |
|  | Teprotumumab | 23.7 (2.7) | 20.4 (2.4) |  |
| Bennedjai et al., 2022 (46) | Tocilizumab | OD: 25 (0.9)  OS: 26 (1.1) | OD: 23 (0.8)  OS: 24 (1.4) | - |
|  | Rituximab | OD: 22 (3.5)  OS: 24 (3.3) | OD:21 (7.3)  OS: 24 (4.1) |  |
| Pan et al., 2022 (47) | Doxycycline | - | -0.18^b^ | 0.37 |
|  | Placebo | - | -0.19^b^ | 0.99 |
| Shen et al., 2022 (48) | MP | 22.3 (2.30) | 21.4 (2.40) | 0.9 |
|  | Reduced MP + MTX | 21.6 (2.10) | 20.8 (2.50) | 1.0 |
|  | MP + MTX | 22.0 (2.70) | 21.6 (2.80) | 0.9 |
| Douglas et al., 2023 (49) | Teprotumumab | 24.6 (3.01) | -2.41^b^ | 0.0004 |
|  | Placebo | 24.0 (2.82) | -0.92^b^ |  |
| Boutzios et al., 2023 (50) | Tocilizumab | - | -2.3^b^ | 0.003 |
| Men et al., 2023 (52) | Teprotumumab | 23.9 | -3.1 (2.4)^b^ | <0.01 |
| Hoang et al., 2024 (53) | Teprotumumab | 23 (24-20) ^a^ | 21 (22.25-17) ^a^ | 0.0001 |
| Rosenblatt et al., 2024 (54) | Teprotumumab | - | -3.36 (1.36) ^b^ | - |
| Habroosh et al. 2024 (56) | Tocilizumab | 24.85 (2.31) | 21.78 (2.18) | 0.000497 |
| Al-Sharif et al., 2025 (58) | Teprotumumab | OD: 21.8 (2.9)  OS: 21.7 (3.3) | OD: 19.1 (2.8)  OS: 19.2 (2.8) | - |
| Farde et al., 2025 (59) | Tocilizumab | OD: 21.07 (3.23)  OS: 20.61 (3.12) | OD: 19.18 (2.52)  OS: 19.95 (2.81) | 0.05 |
| Hiromatsu et al., 2025 (60) | Teprotumumab | 21.07 (2.46) | 24/27^d^ | 0.001 |
|  | Placebo | 20.39 (2.42) | 4/27^d^ |  |

Supplementary Table 6. Results of included studies that investigated changes in proptosis. IVGC: intravenous glucocorticoids; IVMP: intravenous methylprednisolone; MP: methylprednisolone; MTX: methotrexate; OD: oculus dexter (right eye); OR: Orbital Radiotherapy; OS: oculus sinister (left eye); Pred: prednisolone; pts: patients; RTX: rituximab; SD: standard deviation. NOSPECS is an alternative clinical scoring system to Clinical Activity Score.

Supplementary Table 6 addenda

| a | Median (range) |
| --- | --- |
| b | Mean reduction in proptosis (SD) |
| c | No significant treatment effect, no numerical data provided |
| d | Proptosis reduction of 2mm or more |

**Supplementary Table 3 - TRAb reduction**

| Ref | Drug(s) studied | Mean TRAb before (SD) (IU/L) | Mean TRAb after (SD) (IU/L) | P-value |
| --- | --- | --- | --- | --- |
| Kahaly et al., 1996 (22) | IVIG | 104 (28-136) | - | - ^a^ |
|  | Prednisolone | 87 (19-108) | - |  |
| Stan et al., 2015 (33) | RTX | 20 (9-60)^c^ | -0.25^b^ | - |
|  | Placebo | 19.5 (2.2-28.8)^c^ | -4.2^b^ |  |
| Deltour et al., 2018 (36) | RTX | 23.0 (40.2) | 12.3 (25.1) | < 0.001 |
| Insull et al., 2019 (37) | RTX | 6.5 | 2.7 | 0.06 |
| Ceballos-Macias et al., 2020 (39) | Tocilizumab | 291.88 (96.39) | 172.71 (54.01) | 0.001 |
| Perez-Moreiras et al. 2021 (40) | Tocilizumab | 69.0 (87.5) | 17.3 (40.4) | <0.001 |
| Moi et al., 2021 (42) | Tocilizumab | 12.79 (11.92) | 3.20 (4.57) | 0.061 |
| Shen et al., 2022 (48) | MP | 6.2 (2.2-20.5)^c^ | -9.74^b^ | <0.0001 |
|  | Reduced MP + MTX | 9.6 (3.23-21.92)^c^ | -9.16^b^ | 0.0001 |
|  | MP + MTX | 10.1 (3.2-26.8)^c^ | -8.75^b^ | 0.0001 |
| Wang et al., 2023 (51) | RTX | 14.92 (18.48) | 1.58 (2.33) | - |
| Hoang et al., 2024 (53) | Teprotumumab | 3.10 (1.10-12.48) ^c^ | 0.60 (0.00-3.30) ^c^ | 0.0001 |
| Farde et al., 2025 (59) | Tocilizumab | 5.8 | 4.85 | - |

Table 7. Results of studies that investigated reduction in TRAb levels. IVIG: intravenous immunoglobulin; MP: methylprednisolone; MTX: methotrexate; Pred: prednisolone; RTX: rituximab; TRAb: TSH-receptor antibody.

Supplementary Table 7 Addenda

| a | No difference between groups |
| --- | --- |
| b  c | TRAb difference  Median (range) |

**Supplementary Table 4 - B Cell Depletion**

| Ref | Drug(s) studied | B cell count before treatment | No. of patients with decreased B cells after treatment | B cell subtype measured | P-value |
| --- | --- | --- | --- | --- | --- |
| Salvi et al., 2008 (27) | RTX | - | 8/9 | CD19^+^ | - |
|  |  |  |  | CD20^+^ | <0.0001 |
| Silkiss et al., 2010 (29) |  | - | 10/12 | CD19^+^ | - |
| Mitchell et al., 2013 (31) |  | 15.7 | 8/9 | CD19^+^ | - |
| Insull et al., 2019 (37) |  | 0.26 x 10^9^/L (0.097) ^a^ | 0.04 x 10^9^/L (0.020) ^a^ | CD19^+^ | <0.001 |
| Wang et al., 2023 (51) |  | - | 6/6 | CD19^+^  CD20^+^ | - |

Supplementary Table 8. Results of included studies that investigated changes in B cell depletion. CD19^+^: CD19-positive B cell; CD20^+^: CD20-positive B cell; RTX: rituximab.

Supplementary Table 8 Addenda

| a | Mean (Standard deviation) |
| --- | --- |

**Supplementary Table 5 - Quality of Life**

| Ref | Drug studied | Follow-up (weeks) | Mean score after treatment (SD) | | P-value |
| --- | --- | --- | --- | --- | --- |
|  |  |  | Visual Function | Appearance |  |
| Perez-Moreiras et al., 2018 (35) | Tocilizumab | 40 | 46.7^a^ | 33.3^a^ | - |
|  | Placebo |  | 35^a^ | 29.4^a^ |  |
| Douglas et al., 2022 (45) | Teprotumumab | 24 | 28.0 (28.0) | 7.8 (11.5) | - |
|  | Placebo | 24 | 11.7 (22.5) | 15.1 (20.3) | - |
| Pan et al., 2022 (47) | Doxycycline | 4 | 2.89 | 2.70 | 0.90 |
|  |  | 12 | 0.98 | 6.50 |  |
|  | Placebo | 4 | 0.80 | 4.84 | 0.81 |
|  |  | 12 | 0.91 | 7.91 |  |
| Shen et al., 2022 (48) | MP | 12 | 69.3 (27.0) | 69.3 (21.0) | - |
|  | Reduced MP + MTX | 12 | 88.2 (14.1) | 74.8 (25.6) | - |
|  | MP + MTX | 12 | 83.3 (17.4) | 73.4 (21.9) | - |
| Douglas et al., 2023 (49) | Teprotumumab | 24 | 73.9 | 93.4 | - |
|  | Placebo | 24 | - | - | - |
| Matoc et al., 2024 (52) | Doxycycline | 12 | 20 (48.8) | 12 (29.3) | - |
|  | Not treated |  | 14 (34.1) | 18 (43.9) |  |
| Hiromatsu et al., 2025 (60) | Teprotumumab | 24 | 16.22 | 4.39 | 0.022 |
|  | Placebo |  | 19.35 | 8.69 | 0.031 |

Supplementary Table 9. Results of included studies that investigated changes in QoL. *Lower score correlates with less interference with QoL.

Supplementary Table 0 Addenda

| a | Percentage of patients of >8 improvement |
| --- | --- |

**Supplementary Table 6 - Adverse Events**

| Ref | Drug studied | Number of Adverse Events | |
| --- | --- | --- | --- |
|  |  | Minor | Major |
| Antonelli et al., 1992 (20) | Group 1: IVIG + OR | 1 | 0 |
|  | Group 2: IVIG |  |  |
|  | Group 3: MP + OR | 11 | 3 requiring withdrawal from follow-up (diabetes, HTN, hemorrhagic gastritis)  1 major not withdrawn (diabetes) |
| Utech et al., 1995 (21) | Cyclosporine + Pred | HTN, Gingival hyperplasia, Hirsutism  Perioral and digital paraesthesia | 0 |
| Kahaly et al., 1996 (22) | IVIG | 2 | 0 |
|  | Pred | 16 | 2 |
| Ozata et al., 1996 (23) | Octreotide | 4 (mild abdominal discomfort with nausea + vomiting, flatulence, diarrhoea) | 0 |
| Dickinson et al., 2004 (24) | Octreotide | 15 Gastrointestinal upset | 0 |
| Meyer, 2006 (25) | IVMP + Pred + cyclosporine | 30 (Cushing's syndrome, HTN, infection, depression, peptic ulcer) | 1 MI |
| Stan et al., 2006 (26) | Octreotide | 5 (diarrhoea, abdominal pain) | 0 |
| Salvi et al., 2008 (27) | RTX | 3 | 0 |
|  | IVGC | 9 | 0 |
| Khanna et al., 2010 (28) | RTX | 2 (HTN and UTI) | 1 (cardiac arrest after 2nd infusion) |
| Silkiss et al., 2010 (29) | RTX | 0 | 0 |
| Tambe et al., 2010 (30) | IVMP | 0 | 0 |
|  | Pred | 0 | 0 |
| Mitchell et al., 2013 (31) | RTX | 4 (headache, mild myalgia) | 0 |
| Perez-Moreiras et al., 2014 (32) | Tocilizumab | 10 (fatigue, neutropenia, URTI) | 0 |
| Stan et al., 2014 (33) | RTX | 6 | 5 |
| Smith et al.,  2017 (34) | Teprotumumab | 32 (nausea, diarrhoea, hyperthyroidism, muscle spasms) | 5 (IBD, diarrhoea, urinary retention, sepsis, Hashimoto’s encephalopathy) |
| Perez-Moreiras et al., 2014 (35) | Tocilizumab | 56 (infections, headache, gastroenteritis, UTI) | 2 |
| Deltour et al., 2018 (36) | RTX | 0 | 1 (transitory cytokine release syndrome) |
| Insull et al., 2019 (37) | RTX | 4 (infusion-related rash, resolved with 10mg IV chlorphenamine) | 0 |
| Douglas et al., 2020 (38) | Teprotumumab | 35 (muscle spasm, alopecia, fatigue, nausea, diarrhoea, headache) | 1 (infusion reaction) |
| Ceballos-Macias et al., 2020 (39) | Tocilizumab | 2 (fatigue, cough) | 0 |
| Perez-Moreiras et al. 2021 (40) | Tocilizumab | 25 (hypercholesterolaemia, neutropenia, thrombocytopenia, urticaria, cellulitis) | 1 (anaphylaxis) |
| Smith et al., 2021(41) | Tocilizumab | - | - |
| Moi et al., 2021 (42) | Tocilizumab | 5 (neutropenia, skin infection, respiratory tract infection) | 3 (breast cancer, bladder cancer) |
| Ugradar et al., 2022 (43) | Teprotumumab | 0 | 0 |
| Ozello et al., 2022 (44) | Teprotumumab | 3 (hair thinning, tinnitus, myalgia) | 0 |
| Douglas et al., 2022 (45) | Teprotumumab | 32 | 11 |
| Bennedjai et al., 2022 (46) | Tocilizumab | 3 (neutropenia, deranged LFTs, rash) | 0 |
|  | Rituximab | 3 (palpitations, increased chemosis and proptosis) |  |
| Pan et al., 2022 (47) | Doxycycline | 1 mild gastric regurgitation | 0 |
| Shen et al., 2022 (48) | Group 1: reduced IVMP + MTX | 21 | 0 |
|  | Group 2: IVMP + MTX | 38 | 0 |
|  | Group 3: IVMP | 41 | 0 |
| Douglas et al., 2022 (49) | Teprotumumab | 33 | 1 (conductive hearing loss) |
| Boutzios et al., 2023 (50) | Tocilizumab | 4 transient weight gain  2 transient hypercholesterolaemia | 0 |
| Wang et al., 2023 (51) | Rituximab | 1 fatigue | 0 |
| Men et al., 2023 (52) | Teprotumumab | 41 (muscle spasm, hearing impairment, alopecia, fatigue, diarrhoea, hyperglycaemia) | 4 (hearing loss, 2 required early termination) |
| Hoang et al., 2024 (53) | Teprotumumab | 32 (Muscle cramp, hyperglycaemia and hearing problems most common) | 0 |
| Rosenblatt et al. 2024 (54) | Teprotumumab | - | - |
| Matoc et al., 2024 (55) | Doxycycline | 2 (Gastrointestinal discomfort and mild skin sensitivity) | 0 |
| Habroosh et al., 2024 (56) | Tocilizumab | 3 (neutropenia and back pain) | 1 severe leukopenia |
| Lee et al., 2024 (57) | Tocilizumab | 2 (Shingles, headache, dermatitis) | 0 |
| Al-Sharif et al., 2024 (58) | Teprotumumab | - | - |
| Farde et al., 2025 (59) | Tocilizumab | 10 (Transient neutropenia, muscle cramps, skin infection, oral ulcer) | 0 |
| Hiromatsu et al., 2025 (60) | Teprotumumab | 14 (alopecia, muscle spasm, diarrhoea, hyperglycaemia, dry eyes, abdominal pain) | 1 |

Supplementary Table 10. Adverse events reported by each study. AEs: adverse events; GTT: glucose tolerance test; HTN: hypertension; IVGC: IV glucocorticoids; IVIG: intravenous immunoglobulin; IVMP: IV methylprednisolone; MP: methylprednisolone; MTX: methotrexate; OR: Orbital Radiotherapy; Pred: prednisolone; RTX: rituximab.

**
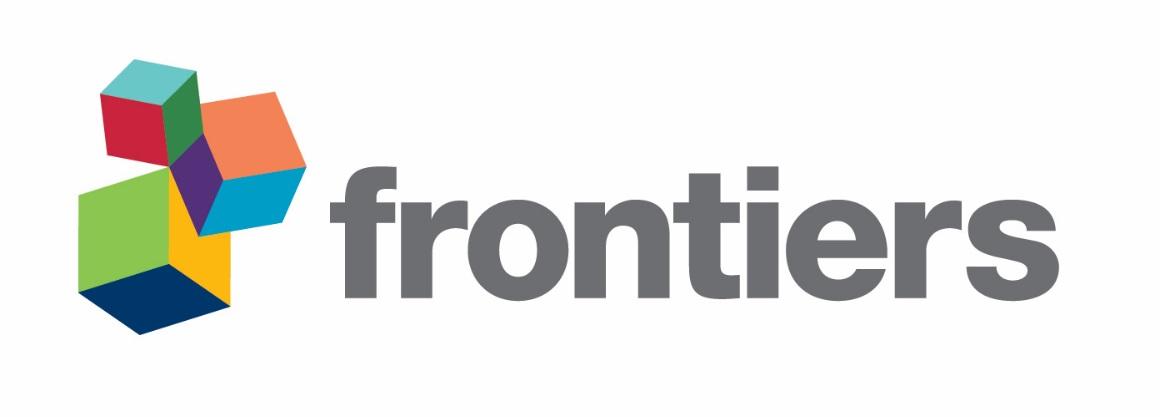
**
